# Conformation–aggregation interplay in the simplest aliphatic ethers probed under high pressure

**DOI:** 10.1107/S2052252523009995

**Published:** 2024-01-01

**Authors:** Natalia Sacharczuk, Anna Olejniczak, Maciej Bujak, Kamil Filip Dziubek, Andrzej Katrusiak, Marcin Podsiadło

**Affiliations:** a Faculty of Chemistry, Adam Mickiewicz University, Uniwersytetu Poznanskiego 8, Poznan 61-614, Poland; b Faculty of Chemistry, University of Opole, Oleska 48, Opole 45-052, Poland; c Institut für Mineralogie und Kristallographie, Universität Wien, Josef-Holaubek-Platz 2, Wien A-1090, Austria; University of Iowa, USA

**Keywords:** conformation–aggregation interplay, aliphatic ethers, *in situ* crystallization, high pressure

## Abstract

The structures of the simplest symmetric primary ethers [(C_*n*_H_2*n*+1_)_2_O, *n* = 1–3] determined under high pressure revealed their conformational preferences and intermolecular interactions. In three new polymorphs of di­ethyl ether (C_2_H_5_)_2_O, high pressure promotes intermolecular CH⋯O contacts and enforces a conversion from the *trans*–*trans* conformer present in the α, β and γ phases to the *trans*–*gauche* conformer, which is higher in energy by 6.4 kJ mol^−1^, in the δ phase. Two new polymorphs of di­methyl ether (CH_3_)_2_O display analogous transformations of the CH⋯O bonds. The crystal structure of di-*n*-propyl ether (C_3_H_7_)_2_O, determined for the first time, is remarkably stable over the whole pressure range investigated from 1.70 up to 5.30 GPa.

## Abstract 2

The most stable *trans*–*trans* conformation of the di­ethyl ether molecule hinders its aggregation due to restricted access to the oxygen atom. This hindrance can be removed by conformational transformation under high pressure.

## 1. Introduction

Pressure can drastically affect the association of molecules, *i.e.* one of the most essential properties of solvents (Boldyreva, 2008 ▸; Fabbiani & Pulham, 2006 ▸; Resnati *et al.*, 2015 ▸). Ethoxy­ethane [di­ethyl ether, (C_2_H_5_)_2_O, hereafter DEE] is a solvent commonly used in chemical practice and has also previously been applied as a general anaesthetic. Raman spectroscopy (Taga *et al.*, 2006 ▸) was used to demonstrate that, in the gaseous and liquid states, the *trans*–*trans* (TT) conformer prevails over the *trans*–*gauche* conformers (TG^+^, TG^−^), which are less stable by *ca* 6.4 kJ mol^−1^. For over a century, DEE has been known to freeze as stable and metastable polymorphs, melting at 157 and 150 K, respectively (Timmermans, 1911 ▸). The melting temperatures, 156.92 K for the stable form and 149.86 K for the metastable form, and the heats of fusion were precisely determined by Counsell *et al.* (1971 ▸). In the stable α phase, determined by André *et al.* (1972 ▸) in the space group *P*2_1_2_1_2_1_ with *Z* = 8, the molecules assume the TT conformation. The crystal structure of the metastable form was not determined, but its vibrational spectra indicated that there are two independent molecules, both in the TT conformation (Durig & Church, 1981 ▸). The existence of the metastable modification and the tendency of DEE to vitrify were explained in terms of the fairly loose structure of the α phase, and hence the low value of stabil­ization energy compared with the amorphous state (André *et al.*, 1972 ▸).

In the literature, there is much less information about the structure, interactions and properties of other aliphatic ethers. Di­methyl ether (DME) freezes at 93 K and its crystal structure was determined by Vojinović *et al.* (2004 ▸). The crystal structure of di-*n*-propyl ether (DPE) had not been determined. It was established that in the structure of tetra­hydro­furan, a cyclic analogue of DEE, molecular aggregation is stabilized by CH⋯O interactions, which are strongly enhanced under high pressure (Dziubek *et al.*, 2010 ▸; Chang *et al.*, 2005 ▸). Such CH⋯O contacts are sterically hindered in the TT conformers, present in the structure of the α phase of DEE. Therefore, acyclic ethers such as DEE and higher ones are expected to change their TT conformations under high pressure. In the simplest ether DME, the molecular structure can be modified owing to the C—O—C angle and the methyl group rotations, whereas the intermolecular interactions can be changed by a rearrangements of molecules. Here, we describe the effect of high pressure on the crystal structure of DEE, and we also studied DME and DPE in order to obtain more general information about the role of molecular structure with respect to the interactions and aggregation for this class of compounds.

## 2. Experimental

**Figure 1:**
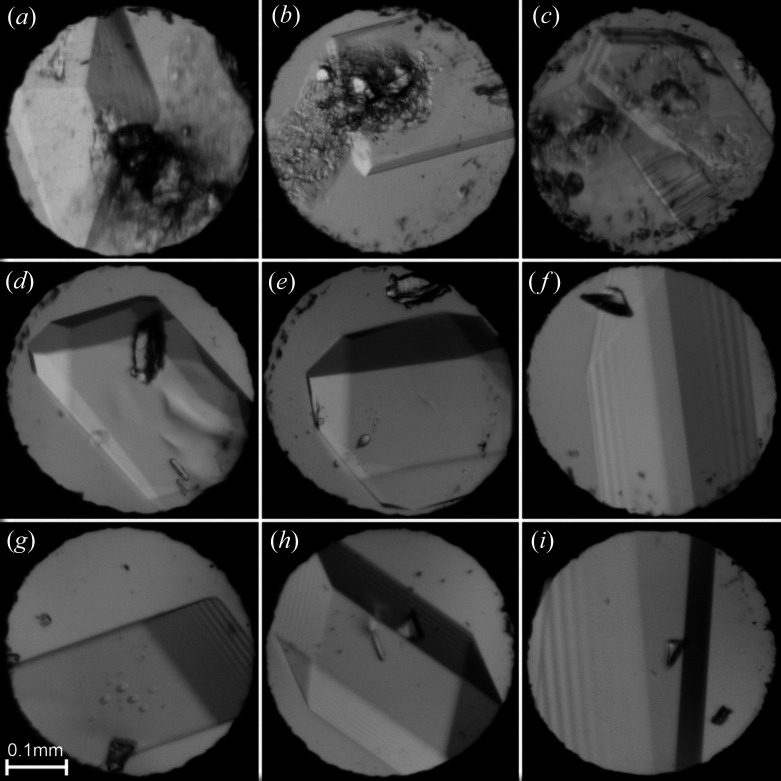
Single crystals grown *in situ* in the DAC. (*a*) β-DME at 3.90 GPa and 349 K, (*b*) γ-DME at 4.50 GPa and 359 K, (*c*) γ-DME at 5.60 GPa and 372 K, (*d*) β-DEE at 1.85 GPa and 327 K, (*e*) β-DEE at 2.45 GPa and 349 K, (*f*) γ-DEE at 2.65 GPa and 340 K, (*g*) δ-DEE at 2.80 GPa and 377 K, (*h*) δ-DEE at 3.45 GPa and 413 K, and (*i*) α-DPE at 2.10 GPa and 344 K.

DME (≥99.9%), DEE (≥99%) and DPE (≥99%), all purchased from Merck, were used as delivered. All three ethers were crystallized *in situ* in a modified Merrill–Bassett diamond-anvil cell (DAC) (Bassett, 2009 ▸). In each experiment, the DAC was equipped with a 0.3 mm-thick steel gasket with a hole 0.4 mm in diameter. At 295 K, DME, DEE and DPE froze at 2.95, 1.50 and 1.35 GPa, respectively, in the form of polycrystalline masses filling the volume of the DAC chamber. Single crystals were obtained under isochoric conditions (Fig. 1 ▸): the DAC containing the squeezed polycrystalline mass of the ether sample was heated with a hot-air gun until all but one grain melted. Then the DAC was slowly cooled to room temperature and the single crystal grew to eventually fill the whole chamber. The temperature inside the DAC was measured using an infrared thermometer. The pressure was calibrated by the ruby fluorescence method (Mao *et al.*, 1986 ▸) before and after the X-ray diffraction measurements using a Photon Control spectrometer with an accuracy of 0.02 GPa. The experimental details and progress in growing the single crystals are shown in Figs. S1–S18 of the supporting information.

The KUMA KM4-CCD diffractometer was used for the high-pressure X-ray diffraction studies. The DAC was centred by the gasket-shadow method (Budzianowski & Katrusiak, 2004 ▸). The *CrysAlisPro* suite was used for data collection, determination of the **UB** matrices and unit-cell parameters, and data reductions (Rigaku Oxford Diffraction, 2019 ▸). For all data, we accounted for the Lorentz, polarization and absorption effects. The programs *SHELXT* (Sheldrick, 2015*a* ▸) and *SHELXL* (Sheldrick, 2015*b* ▸) within the *OLEX2* (Dolomanov *et al.*, 2009 ▸) GUI were used to solve the structures by direct methods and refine the models by full-matrix least-squares on *F* ^2^. Anisotropic displacement parameters were applied for non-hydrogen atoms, but the isotropic thermal parameters were occasionally retained for atoms with unreasonable anisotropic factors or for lower-quality datasets. Hydrogen atoms were located from the molecular geometry, with the C—H distances equal to 0.97 Å (–CH_2_–) or 0.96 Å (–CH_3_) and their *U*_iso_ factors constrained to 1.2 or 1.5 times that of *U*_eq_ of the carriers. The crystal and experimental data are summarized in Table 1 ▸ and Tables S1–S4 of the supporting information.

A conformational analysis of the isolated DEE molecule in the gas phase was performed with the *ab initio* approach of the density functional theory (DFT) with the B3LYP/6–311++g(2d,2p) method using *GAUSSIAN 16W* (Frisch *et al.*, 2016 ▸). The potential energy *E*_p_ map has been created as a function of the torsion angles C2—C1—O1—C3 and C4—C3—O1—C1 with a step of 30° (Dennington *et al.*, 2016 ▸). The methyl hydrogen atoms (constraint AFIX 137) deviate by up to *ca* 8° in the H—C—C—O torsion angles for DEE compared with those obtained from the geometry optimization for the isolated molecule by *GAUSSIAN 16W*. The structure of DEE in the γ phase best agrees with the calculations, and the largest difference was observed for the δ phase.

## 3. Results and discussion

**Figure 2:**
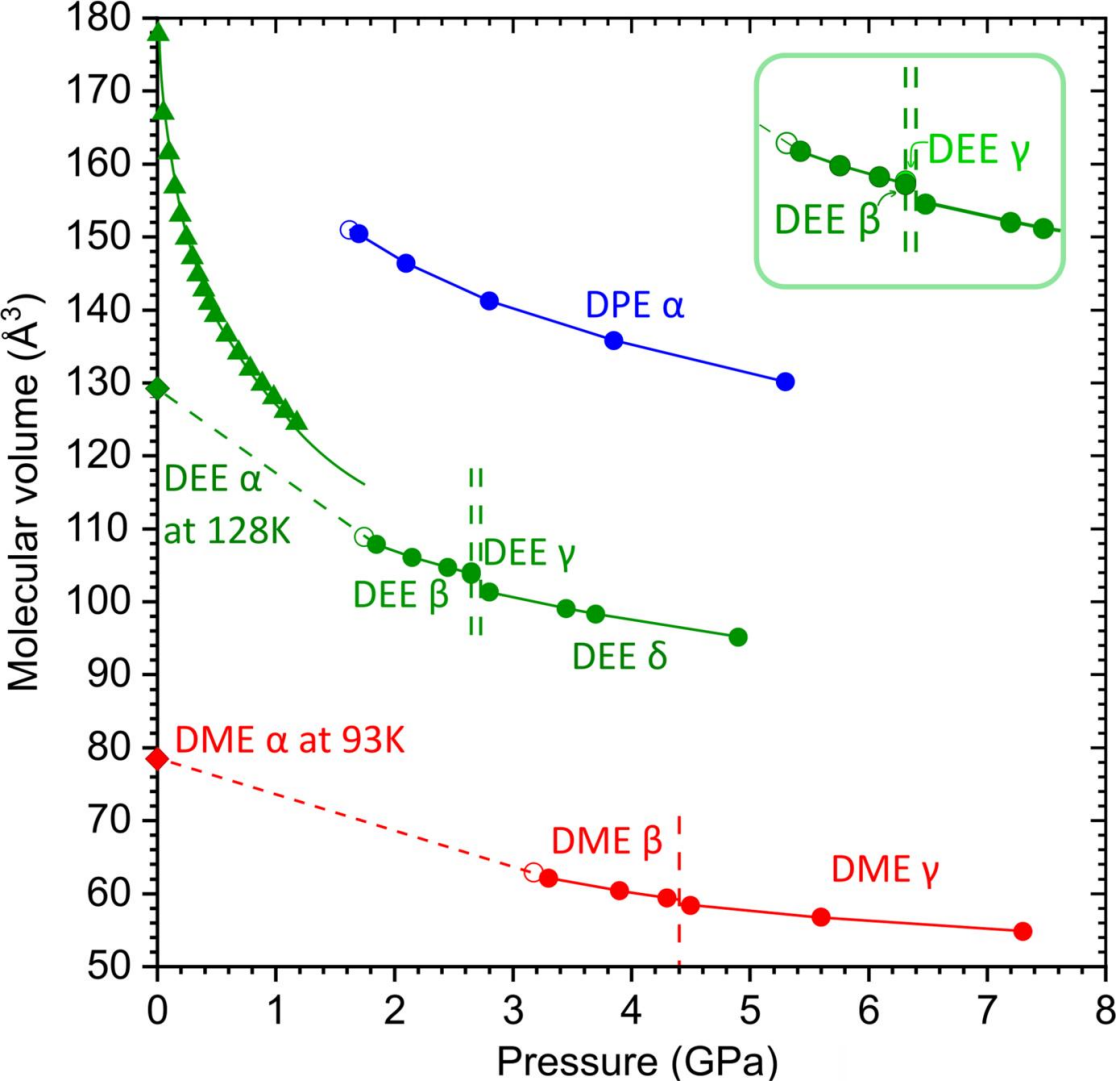
Molecular volume (*V*/*Z*) of DME, DEE and DPE plotted as a function of pressure: solid high-pressure (circles, this work) and low-temperature [diamonds (André *et al.*, 1972 ▸; Vojinović *et al.*, 2004 ▸)] phases as well as liquid DEE [triangles (Bridgman, 1913 ▸)]. Open circles indicate the *V*_m_ values estimated at the freezing-pressure points. The vertical dashed lines mark the solid–solid transition pressure points (this work). The ambient- and high-pressure points at 295 K are joined by dashed lines. The solid lines between points are guides for the eye only. The estimated standard deviations are smaller than the plotted symbols.

The lowest pressure for investigating the structure of the simplest primary ethers was chosen, about 0.3 GPa above their freezing pressure points, to ensure the stability of the single-crystal samples during the X-ray diffraction data collection experiments. The maximum pressure was the result of reaching the mechanical or thermal limitation of the DAC during the procedure to obtain the single crystals. The molecular volumes of the ethers studied as a function of pressure are plotted in Fig. 2 ▸.

We have established that DEE freezes at 1.50 GPa when isothermally compressed at 295 K. Therefore, the single crystal was grown under isochoric conditions from the liquid in a DAC at 1.85 GPa (Fig. 1 ▸). The new β phase, built of TT conformers, is stable up to 2.65 GPa, when the γ phase, also built of the TT conformers, is formed. At even higher pressure, the DEE molecules adopt the TG conformation and the δ phase is formed, investigated between 2.80 and 4.90 GPa (Fig. 2 ▸). In the TG conformers, the reduced steric hindrance around the oxygen atom facilitates the formation of a larger number of CH⋯O contacts. When releasing pressure, the δ phase transforms to the β phase below 2.70 GPa.

In DME, the steric hindrance around the oxygen atom is smaller and the number of H⋯O contacts increases without conformational transformations. At 0.1 MPa and 93 K, DME crystallizes in the centrosymmetric α phase of the tetragonal space group *P*4_2_/*n* (Vojinović *et al.*, 2004 ▸). At 3.30 GPa and 295 K, DME forms the centrosymmetric β phase in the space group *P*2_1_/*c*, and then, with increasing pressure, to 4.40 GPa, it transforms to the γ phase of the space-group symmetry *P* 1 (Fig. 2 ▸). In DPE, the conformation is important for the molecular aggregation. The crystal structure of DPE at 0.1 MPa and low temperature has not yet been reported. At high pressure and 295 K, DPE crystallizes in the centrosymmetric space group *P*2_1_/*c*. It was found that this phase (α phase) is stable from 1.70 to 5.30 GPa at least.

**Figure 3:**
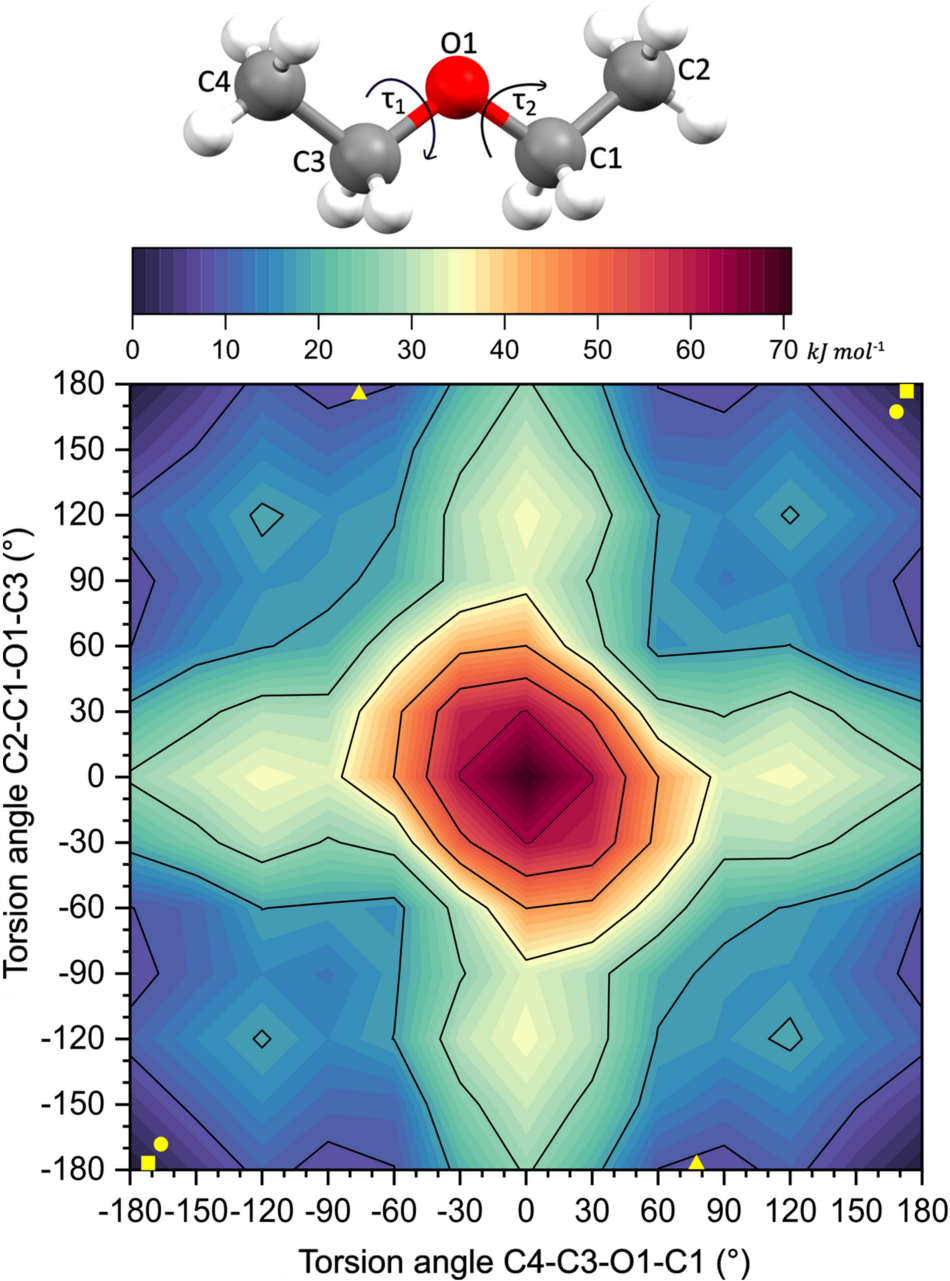
Potential energy (*E*_p_) map as a function of the torsion angles C4—C3—O1—C1 (τ_1_) and C2—C1—O1—C3 (τ_2_) for the isolated DEE molecule, with *E*_p_ = 0 for the TT conformer. The conformers present in the crystalline state for β DEE (square), γ DEE (circle) and δ DEE (triangle) are indicated in yellow.

Our quantum-mechanical computations performed with *Gaussian* (Frisch *et al.*, 2019 ▸) show that the idealized TT conformer (τ_1_ = τ_2_ = 180°) is 8.72 kJ mol^−1^ more stable than the idealized TG conformers (τ_1_ = 180°, τ_2_ = ±60°). This *E*_p_ difference is somewhat larger than that previously determined by the B3LYP/6–311+G** method (Taga *et al.*, 2006 ▸). Owing to the crystal-field effects, the *E*_p_ difference calculated by us between the TT and TG conformers present in the β (τ_1_ = 172.47°, τ_2_ = 179.45°) and δ (τ_1_ = 177.70°, τ_2_ = −77.55°) DEE phases is 6.43 kJ mol^−1^ and between the γ (τ_1_ = τ_2_ = 168.53°) and δ phases it is 5.12 kJ mol^−1^. Therefore, the volume reductions of 2.54 and 2.74 Å^3^ for the β–δ and γ–δ phase transitions, respectively, at 2.70 GPa, associated with the work component of the Gibbs free energies equal to 4.13 and 4.45 kJ mol^−1^, is consistent with the energy gain of the system for a transition involving conformational changes. When assuming the initial density of the liquid at 293 K (0.7134 g cm^−3^), the work performed on the sample to 2.70 GPa amounts to about 59 kJ mol^−1^, which is commensurate with the energy of the *E*_p_ barrier equal to about 11.3 kJ mol^−1^ in the *E*_p_ map in Fig. 3 ▸.

**Figure 4:**
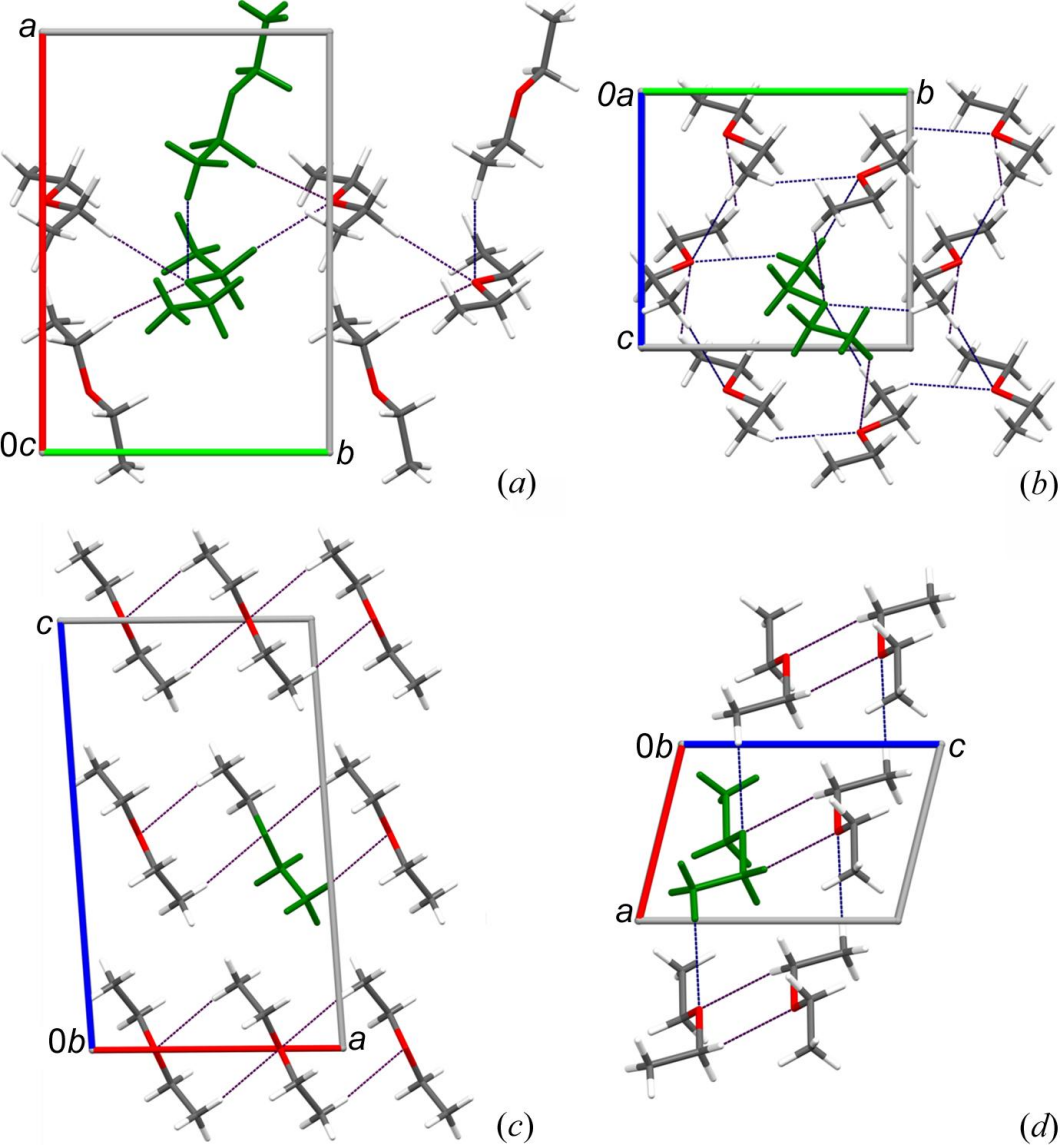
Patterns of the CH⋯O bonds (dotted lines) in the structures of the DEE polymorphs (*a*) α, (*b*) β, (*c*) γ and (*d*) δ. The symmetry-independent structural units (0.5, 1 and 2 molecules) are indicated in green.

According to intermolecular distances, the cohesion forces in DME, DEE and DPE crystals are dominated by CH⋯O bonds (Figs. 4 ▸, 5 ▸ and S19–S26). The approaching hydrogen atoms are roughly (within about 30°) grouped about the directions of the lone-electron pairs of oxygen atoms. For the β and γ phases of DEE, only the methyl hydrogen atoms participate in the hydrogen bonds, whereas in the α and δ phases of DEE, there are both methyl and methyl­ene hydrogen donors. The CH⋯O bonds aggregate the molecules into different and characteristic architectures of rings (α DME), chains (α DEE, γ DEE, α DPE), ribbons (δ DEE), sheets (β DME, β DEE) and a three-dimensional pattern (γ DME). In DME, the number of CH⋯O contacts that are shorter than the sum of the van der Waals radii (Bondi, 1964 ▸) increases with pressure, hence the three-dimensional aggregation patterns are promoted. This relation does not apply for the DEE polymorphs.

The four phases of DEE clearly reveal the systematic transformation of patterns of intermolecular interactions at high pressure. The initial compression of H⋯H contacts and the small compression of H⋯O contacts between the α, β and γ phases are reversed in the δ phase, where the conformational change increases the access to the oxygen atom (Fig. S20). It promotes the formation of CH⋯O contacts at high pressure. The CH⋯O bonded molecules in the β, γ and δ phases are attracted with intermolecular interaction energies of about −12.0, −11.4 and −9.9 kJ mol^−1^, respectively compared with about −4 kJ mol^−1^ for the molecules with H⋯H contacts only (Gavezzotti, 1994 ▸; Gavezzotti & Filippini, 1994 ▸).

## 4. Conclusions

The interplay of preferences for the CH⋯O bond and low-*E*_p_ conformation govern the aggregation in solid phases of simple aliphatic ethers. We have found six new polymorphs: the β and γ phases of DME; the β, γ and δ phases of DEE; and the α phase of DPE. The conformational conversions can regulate access to the oxygen atom, and in this way can increase the number of stronger CH⋯O bonds and reduce the number of weak H⋯H contacts. Consequently, we observed a higher compressibility of CH⋯O distances in δ DEE compared with the compressibility of H⋯H within this phase. Though high pressure has proved to be a useful tool for inducing conformational changes in simple molecular compounds, it also shows the energetic landscape of thermally activated conformational conversions in liquids. Cohesion forces, molecular conformations and aggregation in the crystal structures of simple ethers still require further studies by theoretical methods. There are also asymmetric ethers, which can provide additional information about the structure–property relations of ethers, however their applications and availability are limited.

## Supplementary Material

Crystal structure: contains datablock(s) global, dimethyl_ether_phase_beta_3_30GPa, dimethyl_ether_phase_beta_3_90GPa, dimethyl_ether_phase_beta_4_30GPa, dimethyl_ether_phase_gamma_4_50GPa, dimethyl_ether_phase_gamma_5_60GPa, dimethyl_ether_phase_gamma_7_30GPa, diethyl_ether_phase_beta_1_85GPa, diethyl_ether_phase_beta_2_15GPa, diethyl_ether_phase_beta_2_45GPa, diethyl_ether_phase_beta_2_65GPa, diethyl_ether_phase_gamma_2_65GPa, diethyl_ether_phase_delta_2_80GPa, diethyl_ether_phase_delta_3_45GPa, diethyl_ether_phase_delta_3_70GPa, diethyl_ether_phase_delta_4_90GPa, dipropyl_ether_phase_alpha_1_70GPa, dipropyl_ether_phase_alpha_2_10GPa, dipropyl_ether_phase_alpha_2_80GPa, dipropyl_ether_phase_alpha_3_85GPa, dipropyl_ether_phase_alpha_5_30GPa. DOI: 10.1107/S2052252523009995/lq5055sup1.cif

Supporting figures and tables. DOI: 10.1107/S2052252523009995/lq5055sup2.pdf

CCDC references: 2282455, 2282456, 2282457, 2282458, 2282459, 2282460, 2282461, 2282462, 2282463, 2282464, 2282465, 2282466, 2282467, 2282468, 2282469, 2282470, 2282471, 2282472, 2282473, 2282474

## Figures and Tables

**Figure 5:**
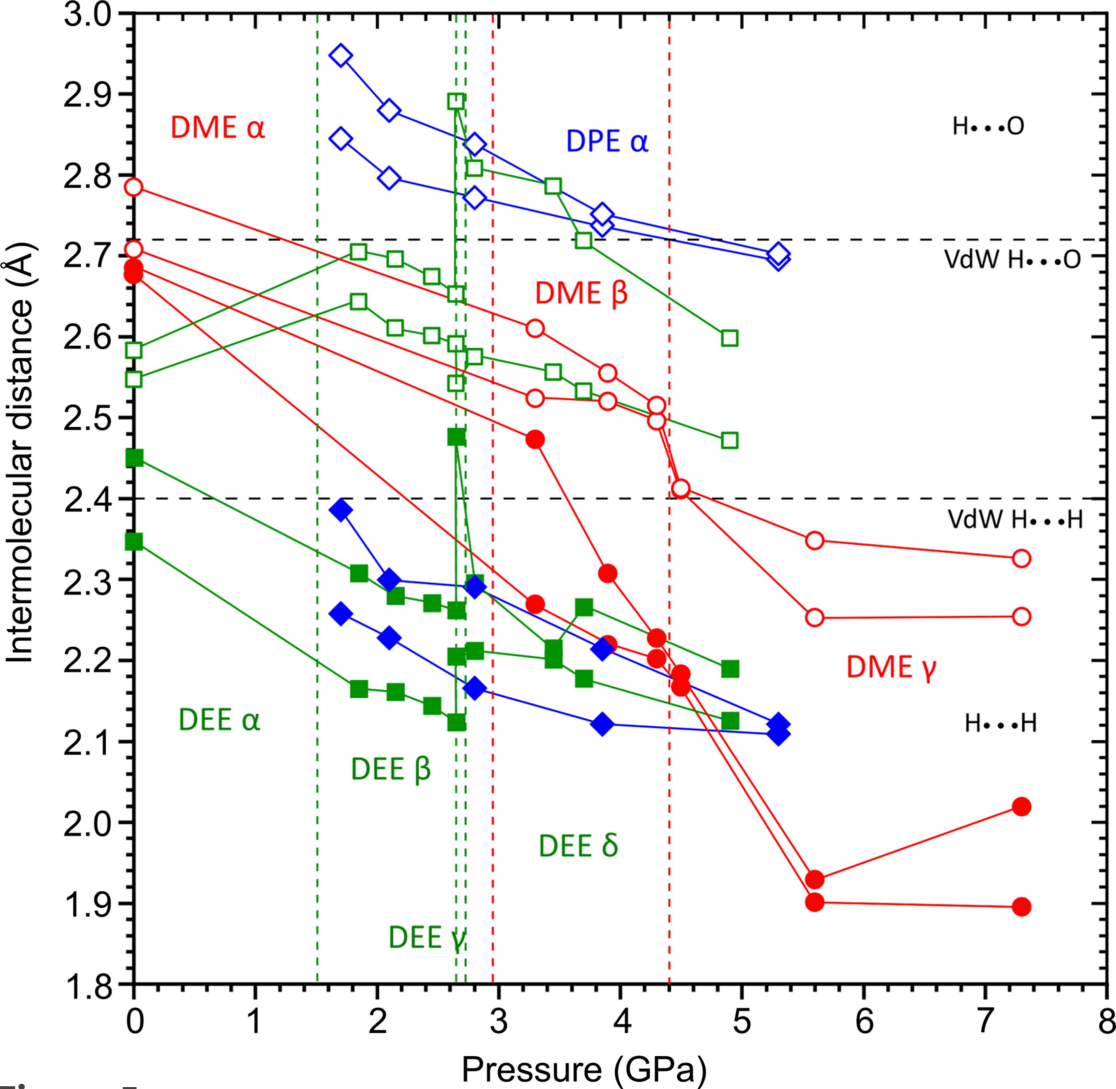
Evolution of the intermolecular distances for the different phases of DME (red), DEE (green) and DPE (blue) with pressure. The two shortest distances for the two types of interactions are presented: full shapes represent H⋯H and empty shapes represent H⋯O distances. The black horizontal lines show the sum of the van der Waals radii (Bondi, 1964 ▸) of hydrogen and oxygen (2.72 Å) and of hydrogen and hydrogen (2.4 Å); the vertical dashed lines mark the limits of the pressure stability ranges of individual crystal phases. The estimated standard deviations are smaller than the plotted symbols.

**Table 1:** Selected crystal data of high-pressure phases of DME, DEE and DPE at 295 K (*cf* detailed data of all 20 determinations in Tables S1–S4)

	β DME	γ DME	β DEE	γ DEE	δ DEE	α DPE
*P* (GPa)	3.30 (2)	4.50 (2)	1.85 (2)	2.65 (2)	2.80 (2)	1.70 (2)
Space group	*P*2_1_/*c*	*P* 1	*P*2_1_/*c*	*I*2/*a*	*P* 1	*P*2_1_/*c*
*a* (Å)	5.5541 (4)	4.3394 (12)	6.8268 (3)	7.7073 (12)	5.1196 (4)	9.416 (4)
*b* (Å)	6.6179 (11)	8.414 (2)	8.1428 (17)	4.0885 (4)	5.6659 (10)	4.1817 (3)
*c* (Å)	6.964 (3)	12.821 (6)	7.7731 (3)	13.233 (2)	7.2999 (4)	15.579 (7)
α (°)	90	90.55 (4)	90	90	97.275 (8)	90
β (°)	103.84 (2)	93.89 (6)	93.443 (4)	93.793 (16)	102.728 (6)	101.23 (5)
γ (°)	90	90.83 (2)	90	90	96.747 (10)	90
*V* (Å^3^)	248.56 (12)	467.0 (3)	431.32 (9)	416.07 (10)	202.56 (4)	601.7 (4)
*Z*, *Z*′	4, 1	8, 4	4, 1	4, 0.5	2, 1	4, 1
*D_x_* (g cm^−3^)	1.231	1.311	1.141	1.183	1.215	1.128
*R*_1_ [*F* ^2^ > 2σ(*F* ^2^)]	0.0708	0.0554	0.0353	0.0371	0.0388	0.0455
