# Supplementary material for: Conformation–aggregation interplay in the simplest aliphatic ethers probed under high pressure
Source: IUCrJ. 2024 Jan 1;11(Pt 1):57–61. doi: 10.1107/S2052252523009995 (PMC10833391; doi:10.1107/S2052252523009995)
Supplement: Supplementary file 2 [file m-11-00057-sup2.pdf]

IUCrJ

Volume 11 (2024)

Supporting information for article:

Conformation-aggregation interplay in the simplest aliphatic ethers probed under high pressure

Natalia Sacharczuk, Anna Olejniczak, Maciej Bujak, Kamil Filip Dziubek, Andrzej Katrusiak and Marcin Podsiadło

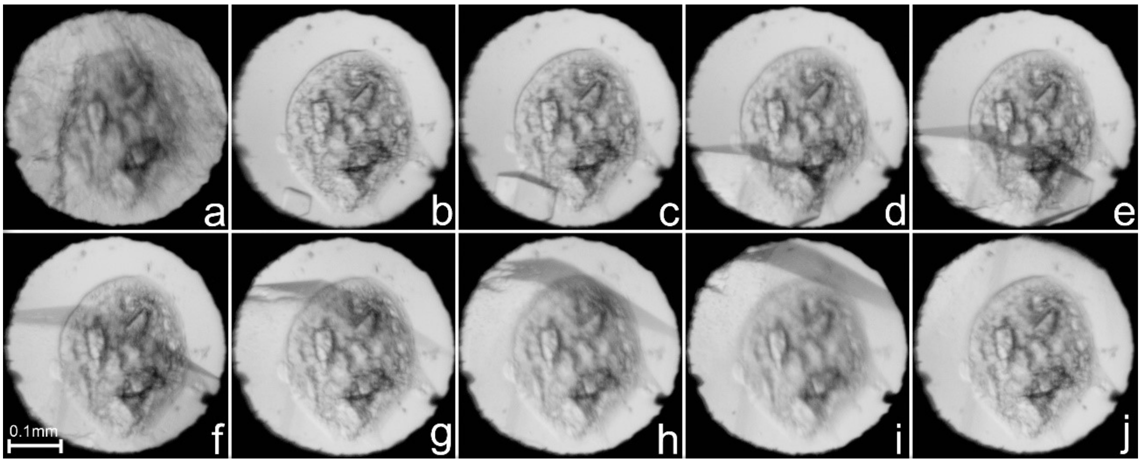

Figure S1. Stages of the dimethyl ether (DME) phase β single-crystal growth inside the DAC chamber: (a) polycrystalline mass grown isothermally at 295 K; (b) one crystal seed at 325 K; (c-i) the single crystal cooled to 311 K and (j) filling the whole volume of the DAC chamber at 295 K and 3.30 GPa. The ruby chips, for pressure calibration, lie in the central part of the DAC.

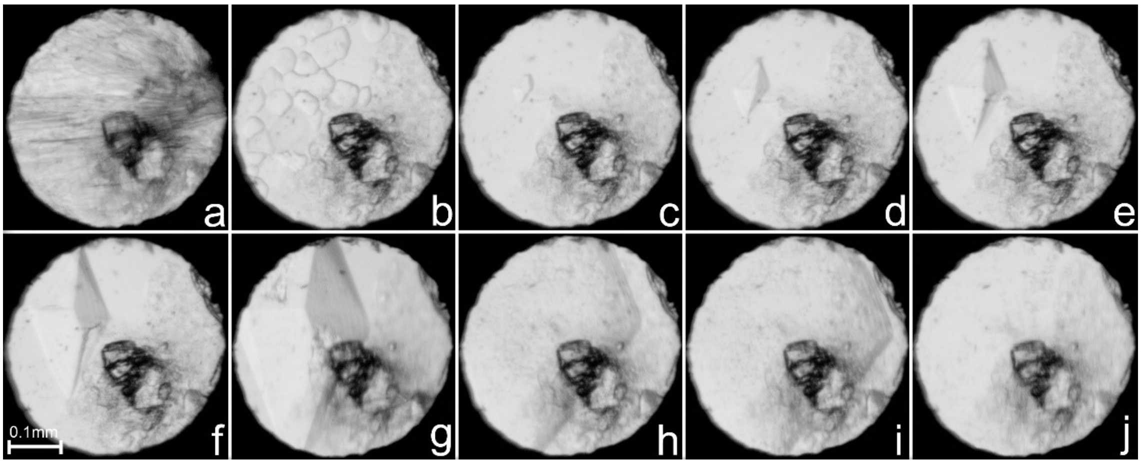

Figure S2. Stages of the DME phase β single-crystal growth inside the DAC chamber: (a) polycrystalline mass grown isothermally at 295 K; (b) polycrystal–liquid equilibrium at 351 K; (c) one crystal seed at 353 K; (d-i) the single crystal cooled to 347 K and (j) filling the DAC chamber at 295 K and 3.90 GPa. The ruby chip, for pressure calibration, is located in the central part of the DAC.

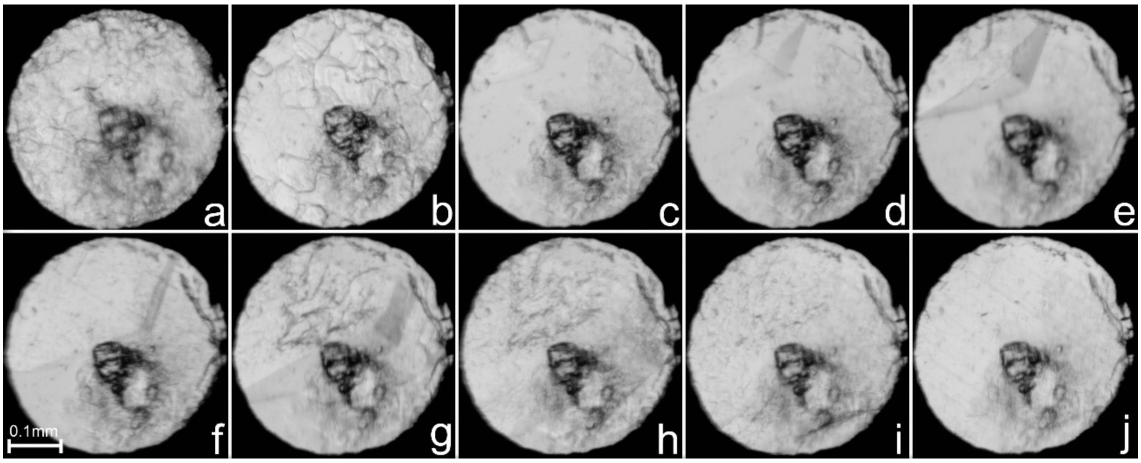

Figure S3. Stages of the DME phase β single-crystal growth inside the DAC chamber: (a) polycrystalline mass grown isothermally at 295 K; (b) polycrystal–liquid equilibrium at 360 K; (c) one crystal seed at 361 K; (d-i) the single crystal cooled to 346 K and (j) filling the whole volume of the DAC chamber at 295 K and 4.30 GPa. The ruby chip, for pressure calibration, is placed in the central part of the DAC.

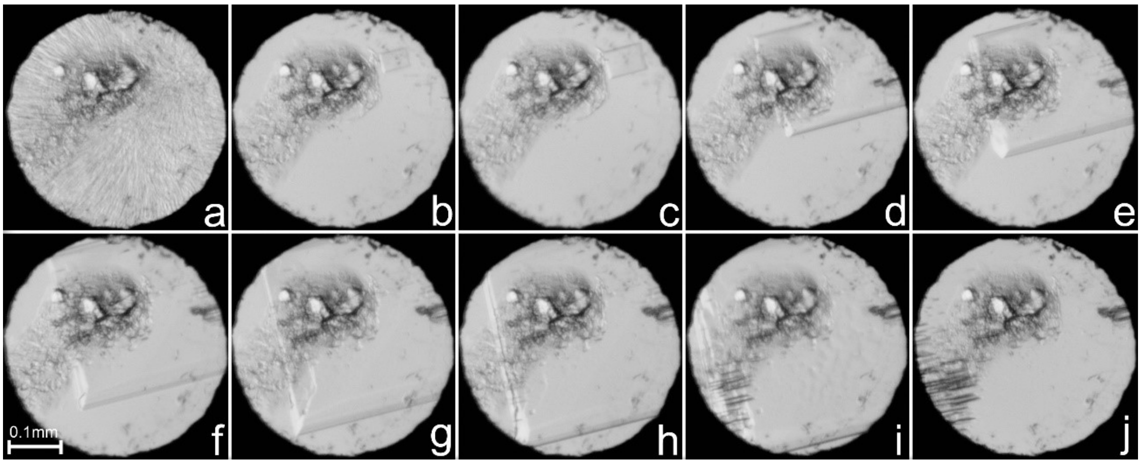

Figure S4. Stages of the DME phase γ single-crystal growth inside the DAC chamber: (a) polycrystal mass grown isothermally at 295 K; (b) one crystal seed at 361 K; (c-i) the single crystal cooled to 351 K and (j) filling the DAC chamber at 295 K and 4.50 GPa. The ruby chips, for pressure calibration, lie in the upper part of the DAC.

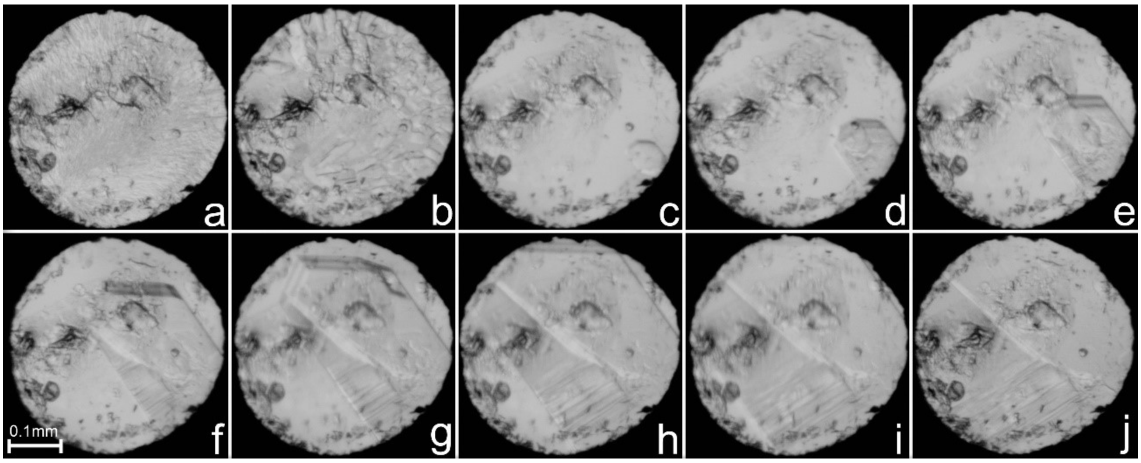

Figure S5. Stages of the DME phase γ single-crystal growth inside the DAC chamber: (a) polycrystalline mass grown isothermally at 295 K; (b) polycrystal–liquid equilibrium at 372 K; (c) one crystal seed at 374 K; (d-i) the single crystal cooled to 369 K and (j) filling the whole volume DAC chamber at 295 K and 5.60 GPa. The ruby chips, for pressure calibration, are placed in the central and left part of the DAC.

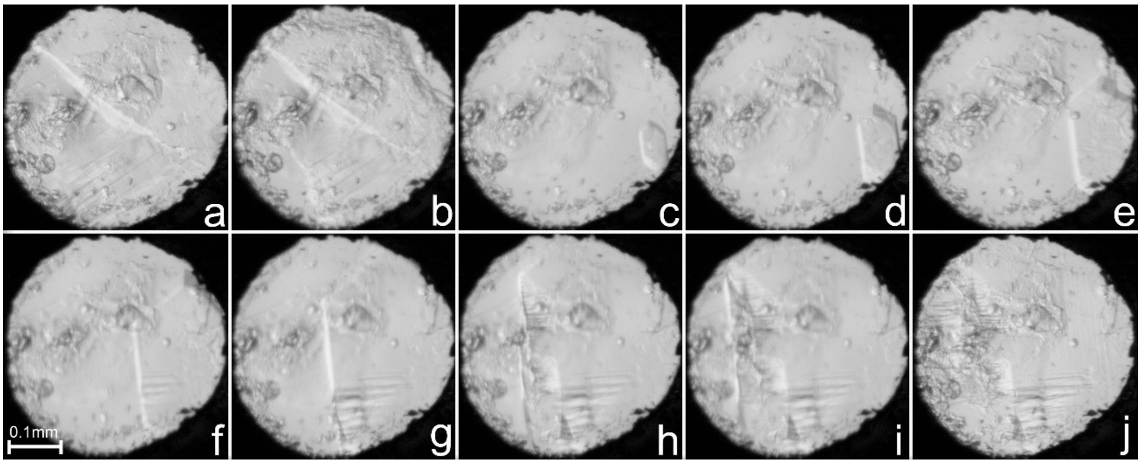

Figure S6. Stages of the DME phase γ single-crystal growth inside the DAC chamber: (a) polycrystalline mass grown isothermally at 295 K; (b) polycrystal–liquid equilibrium at 396 K; (c) one crystal seed at 399 K; (d-i) the single crystal cooled to 394 K and (j) filling the whole volume DAC chamber at 295 K and 7.30 GPa. The ruby chips, for pressure calibration, lie in the central and left part of the DAC.

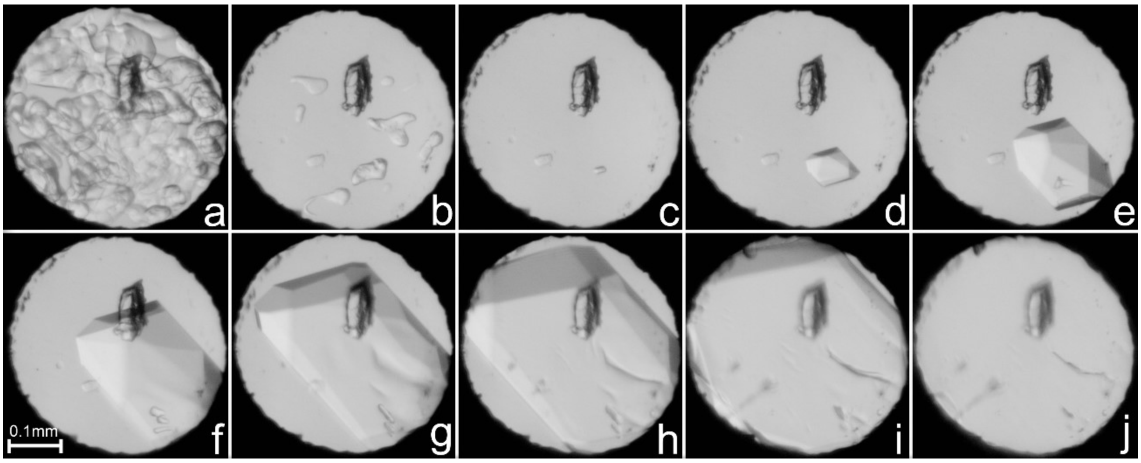

Figure S7. Stages of the diethyl ether (DEE) phase β single-crystal growth inside the DAC chamber: (a) polycrystalline mass grown isothermally at 295 K; (b) polycrystal–liquid equilibrium at 344 K; (c) one crystal seed at 345 K; (d-i) the single crystal cooled to 317 K and (j) filling the whole volume of DAC chamber at 295 K and 1.85 GPa. The ruby chip, for pressure calibration, is placed in the central part of the DAC chamber.

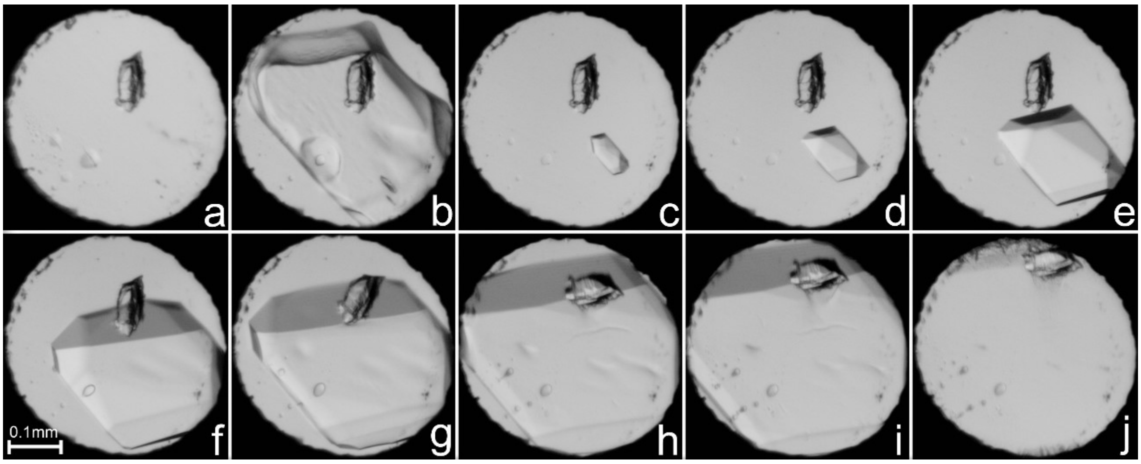

Figure S8. Stages of the DEE phase β single-crystal growth inside the DAC chamber: (a) single-crystal–liquid equilibrium at 308 K; (b) single-crystal–liquid equilibrium at 348 K; (c) one crystal seed at 352 K; (d-i) the single crystal cooled to 331 K and (j) filling the DAC chamber at 295 K and 2.15 GPa. The ruby chip, for pressure calibration, lie in the central part (a-f) and then it is moved to the upper part (g-j) of the DAC.

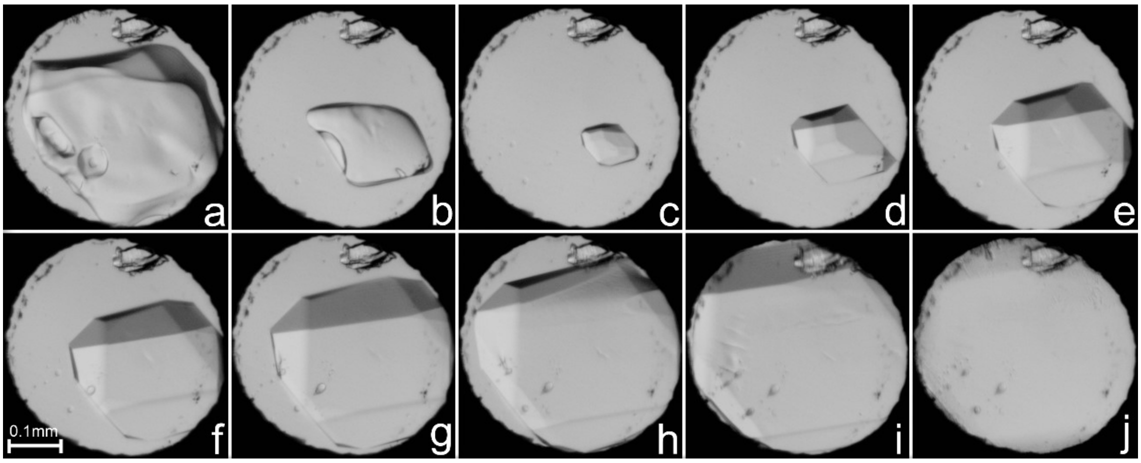

Figure S9. Stages of the DEE phase β single-crystal growth inside the DAC chamber: (a) single-crystal–liquid equilibrium at 360 K; (b) single-crystal–liquid equilibrium at 361 K; (c) one crystal seed at 362 K; (d-i) the single crystal cooled to 338 K and (j) filling the whole volume of DAC chamber at 295 K and 2.45 GPa. The ruby chip for pressure calibration is located in the upper part of the DAC.

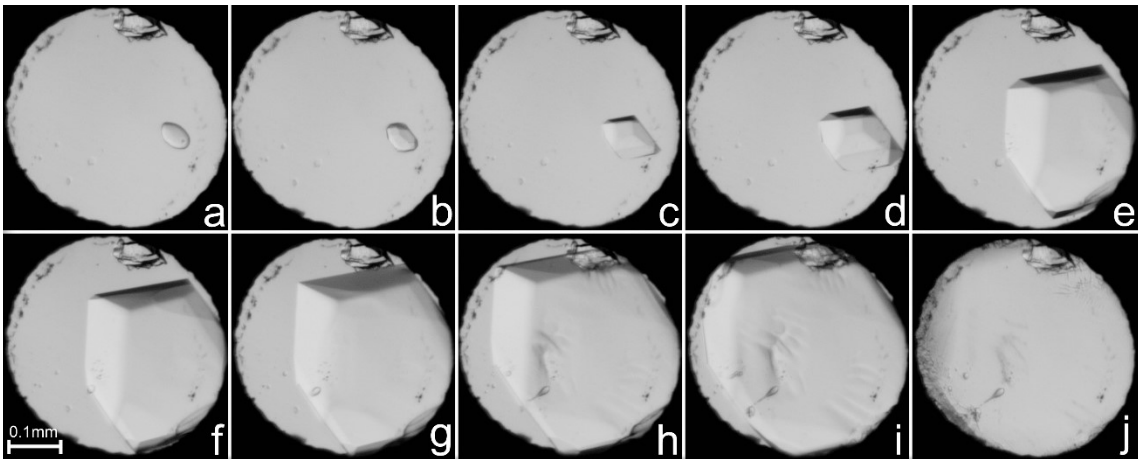

Figure S10. Stages of the DEE phase β single-crystal growth inside the DAC chamber: (a) one crystal seed at 368 K; (b-i) the single crystal cooled to 345 K and (j) filling the DAC chamber at 295 K and 2.65 GPa. The ruby chip, for pressure calibration, lies in the upper part of the DAC.

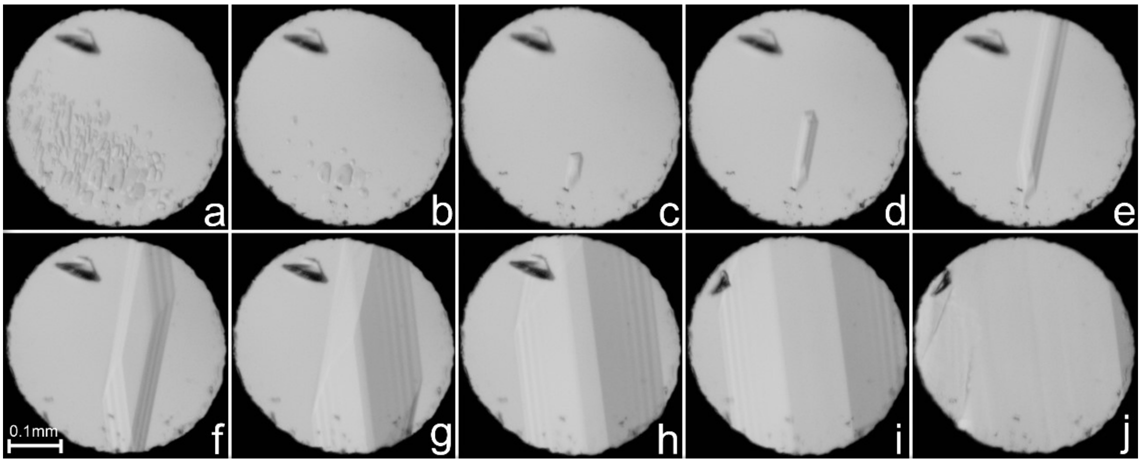

Figure S11. Stages of the DEE phase γ single-crystal growth inside the DAC chamber: (a) polycrystal–liquid equilibrium at 361 K; (b) polycrystal–liquid equilibrium at 363 K; (c) one crystal seed at 359 K; (d-i) the single crystal cooled to 331 K and (j) filling the whole volume of DAC chamber at 295 K and 2.65 GPa. The ruby chip, for pressure calibration, lies in the upper part of the DAC.

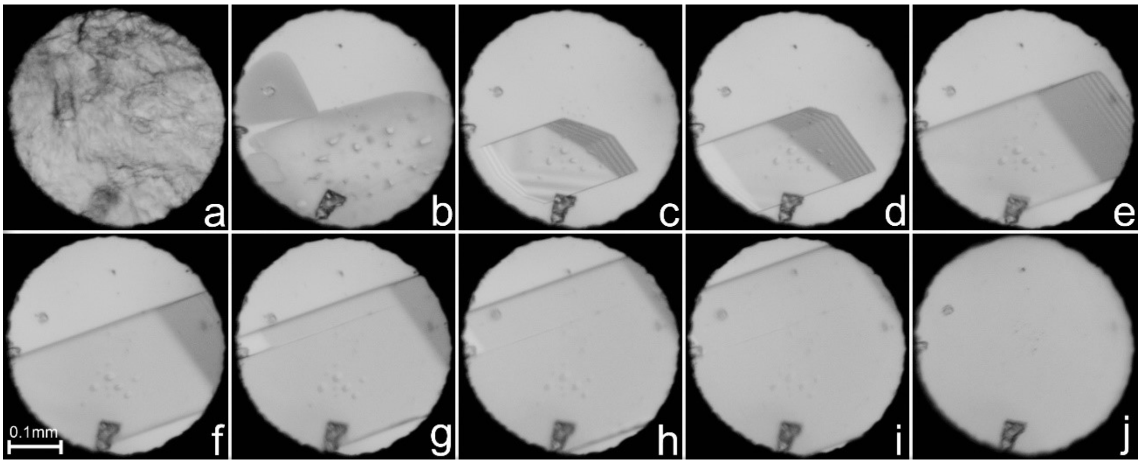

Figure S12. Stages of the DEE phase δ single-crystal growth inside the DAC chamber: (a) polycrystalline mass grown isothermally at 295 K; (b) three single-crystals–liquid equilibrium at 405 K; (c) one crystal seed at 391 K; (d-i) the single crystal cooled to 341 K and (j) filling the DAC chamber at 295 K and 2.80 GPa. The ruby chip, for pressure calibration, lies in the bottom part of the DAC.

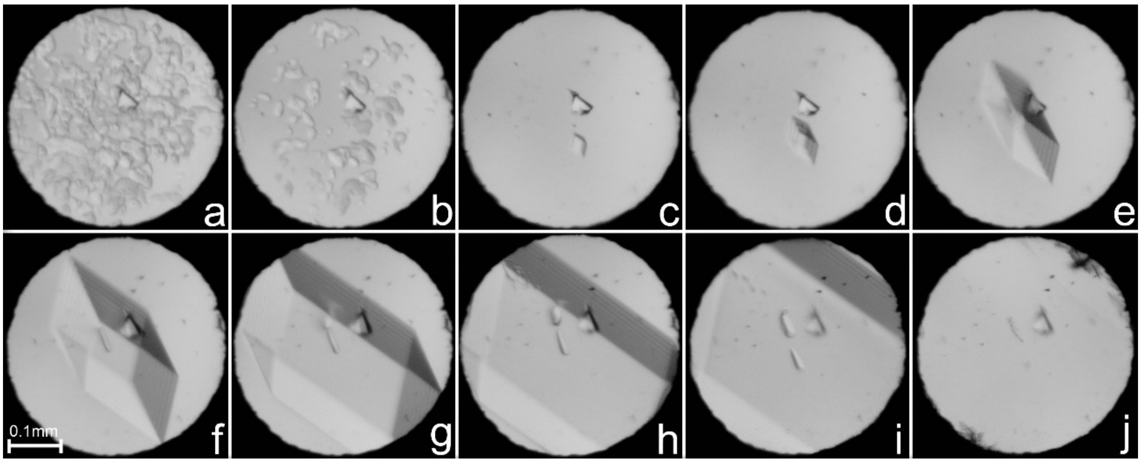

Figure S13. Stages of the DEE phase δ single-crystal growth inside the DAC chamber: (a) polycrystal–liquid equilibrium at 443 K; (b) polycrystal–liquid equilibrium at 444 K; (c) one crystal seed at 445 K; (d-i) the single crystal cooled to 387 K and (j) filling the whole volume of DAC chamber at 295 K and 3.45 GPa. The ruby chip, for pressure calibration, lies in the central part of the DAC.

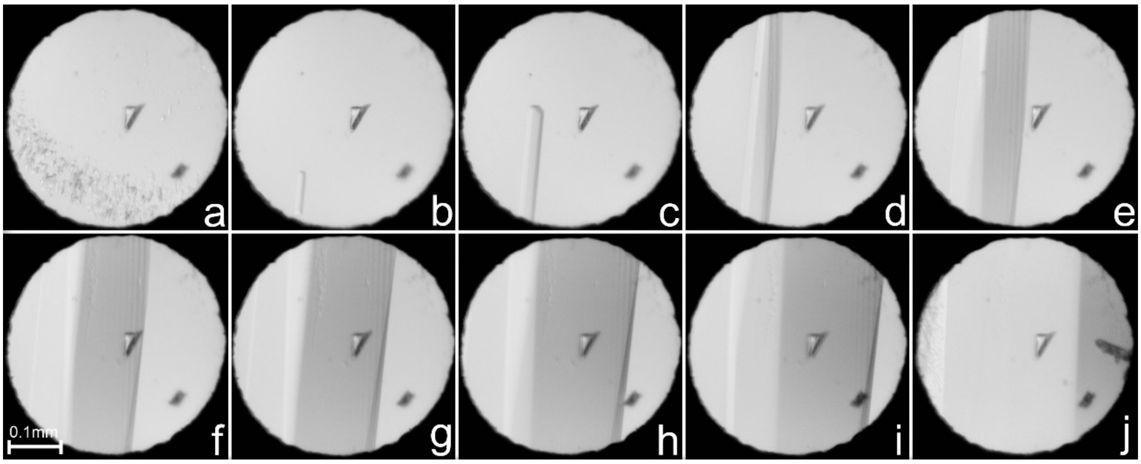

Figure S14. Stages of the dipropyl ether (DPE) phase α single-crystal growth inside the DAC chamber: (a) polycrystal–liquid equilibrium at 334 K; (b) one crystal seed at 334 K; (c-i) the single crystal cooled to 306 K and (j) filling the DAC chamber at 295 K and 1.70 GPa. The ruby chip, for pressure calibration, lies in the central part of the DAC.

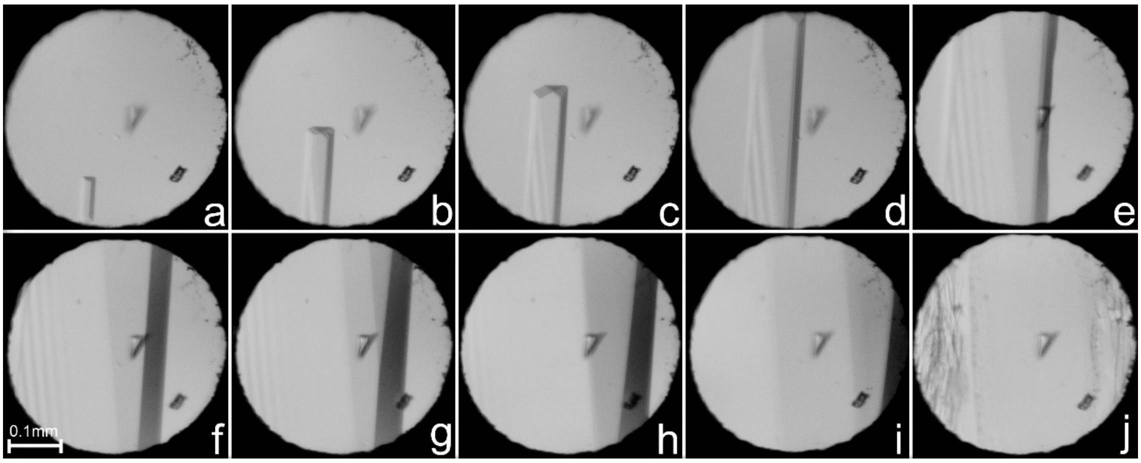

Figure S15. Stages of the DPE phase α single-crystal growth inside the DAC chamber: (a) one crystal seed at 356 K; (b-i) the single crystal cooled to 318 K and (j) filling the whole volume of DAC chamber at 295 K and 2.10 GPa. The ruby chip, for pressure calibration, lies in the central part of the DAC.

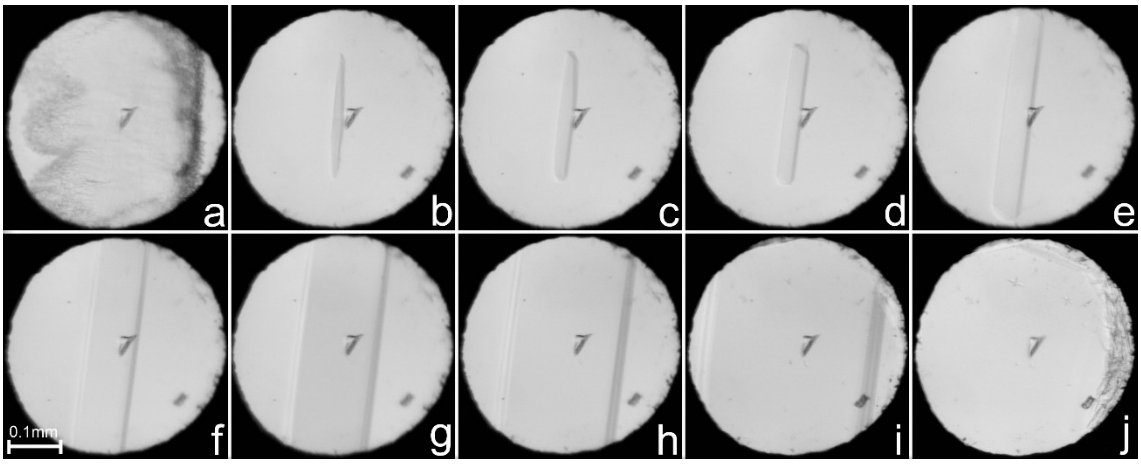

Figure S16. Stages of the DPE phase α single-crystal growth inside the DAC chamber: (a) polycrystal–liquid equilibrium at 393 K; (b) one crystal seed at 396 K; (c-i) the single crystal cooled to 356 K and (j) filling the DAC chamber at 295 K and 2.80 GPa. The ruby chip for pressure calibration lies in the central part of the DAC.

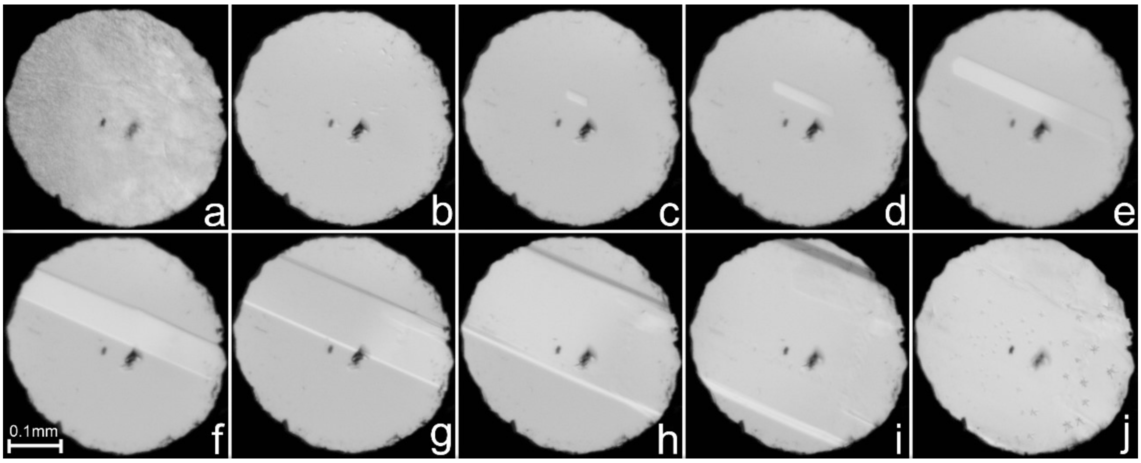

Figure S17. Stages of the DPE phase α single-crystal growth inside the DAC chamber: (a) polycrystal–liquid equilibrium at 295 K; (b) polycrystal–liquid equilibrium at 437 K; (c) one crystal seed at 433 K; (d-i) the single crystal cooled to 401 K and (j) filling the whole volume of DAC chamber at 295 K and 3.85 GPa. The ruby chip, for pressure calibration, lies in the central part of the DAC.

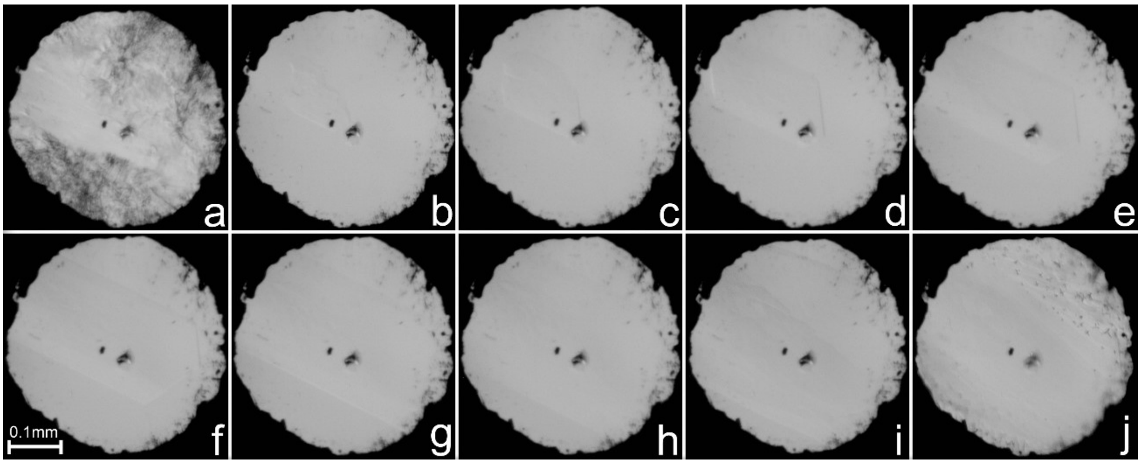

Figure S18. Stages of the DPE phase α single-crystal growth inside the DAC chamber: (a) polycrystal–liquid equilibrium at 295 K; (b) one crystal seed at 483 K; (c-i) the single crystal cooled to 424 K and (j) filling the DAC chamber at 295 K and 5.30 GPa. The ruby chip, for pressure calibration, lies in the central part of the DAC.

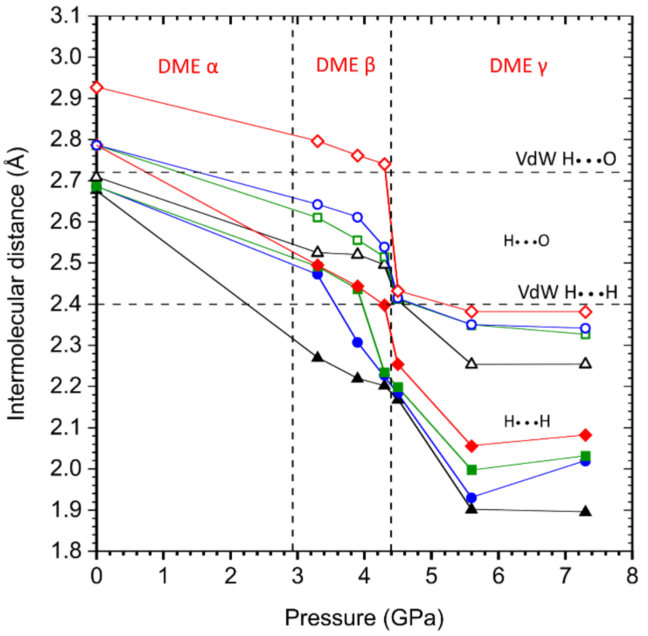

Figure S19. Intermolecular distances plotted as a function of pressure in DME. Four shortest distances for two types of interactions are presented: full shapes represent H···H whereas empty shapes depict the H···O distances. The black horizontal lines show the sum of the van der Waals radii of H and O of 2.72 Å, of H and H of 2.4 Å. The estimated standard deviations are smaller than the plotted symbols.

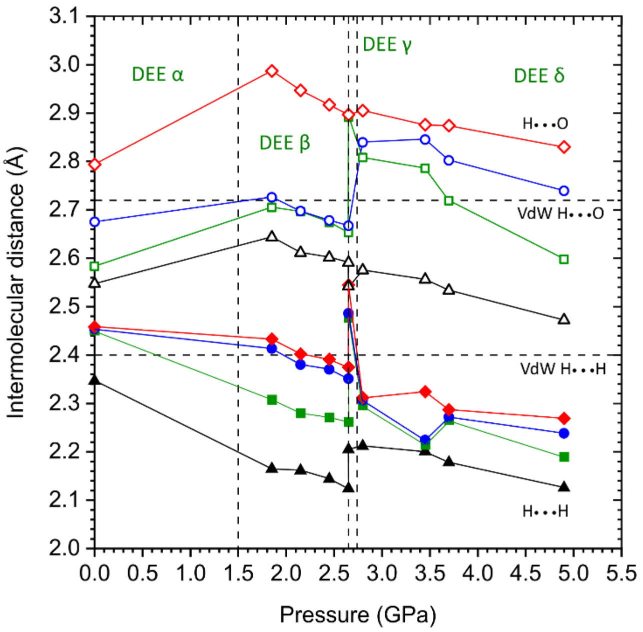

Figure S20. Intermolecular distances plotted as a function of pressure in DEE. Four shortest distances for two types of interactions are presented: full shapes represent H···H whereas empty shapes depict the H···O distances (only two shortest H···O distances for DEE γ are indicated). The black horizontal lines show the sum of the van der Waals radii of H and O of 2.72 Å, and of H and H of 2.4 Å. The estimated standard deviations are smaller than the plotted symbols.

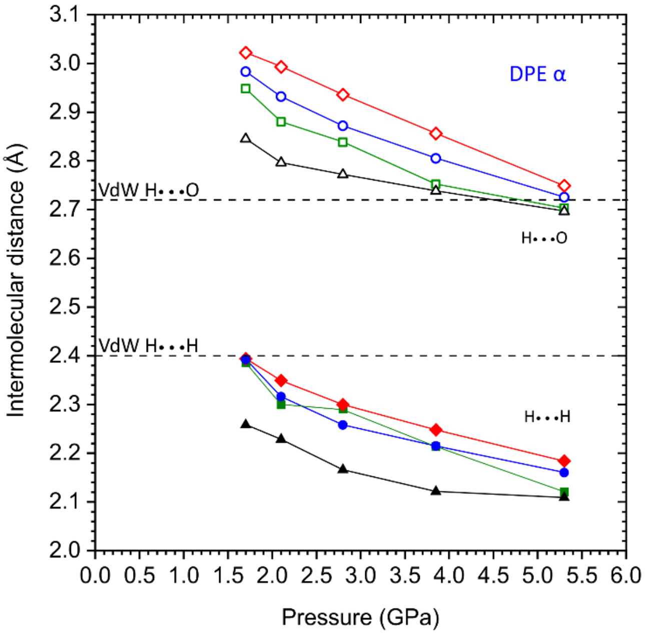

Figure S21. Intermolecular distances plotted as a function of pressure in DPE. Four shortest distances for two types of interactions are presented: full shapes represent H···H whereas empty shapes depict the H···O distances. The black horizontal lines show the sum of the van der Waals radii of H and O of 2.72 Å, and of H and H of 2.4 Å. The estimated standard deviations are smaller than the plotted symbols.

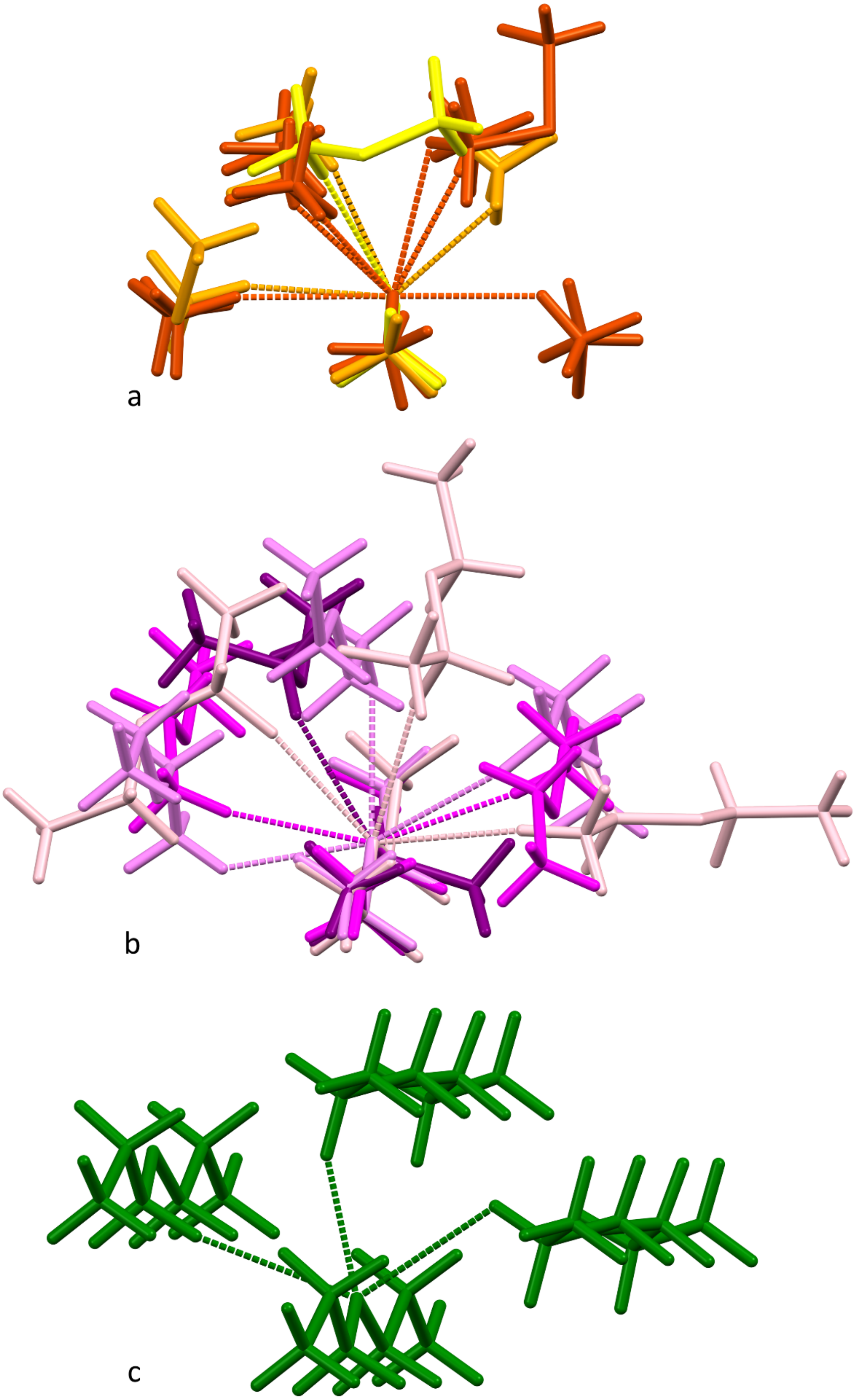

Figure S22. The shortest intermolecular CH···O interactions in: (a) DME phases α (yellow), β (orange) and γ (brown); (b) DEE phases α (pink), β (violet), γ (magenta) and δ (purple) and (c) DPE phase α (green).

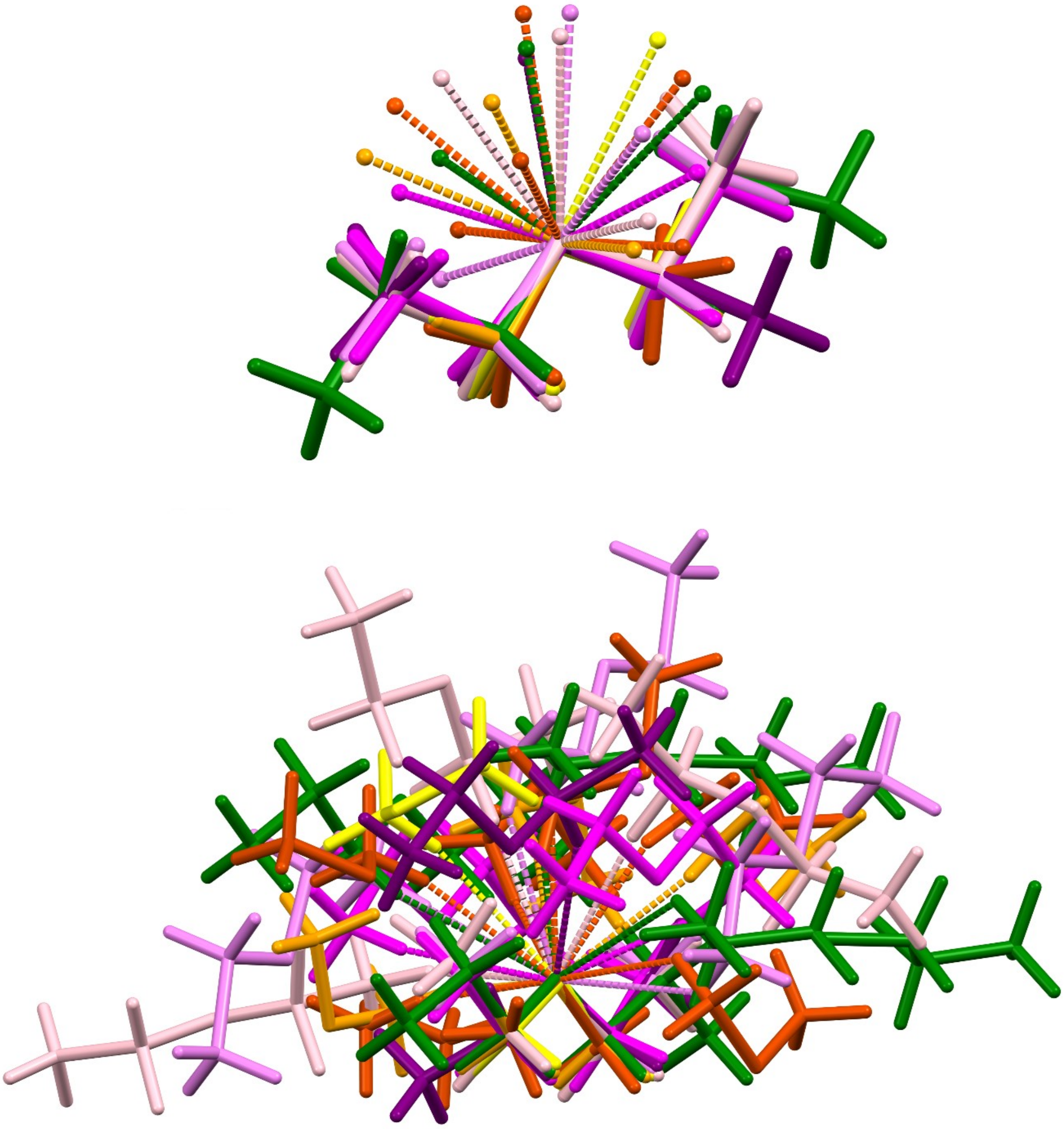

Figure S23. The shortest intermolecular CH···O interactions in the superimposed molecules of DME: phases α (yellow), β (orange), γ (brown); DEE phases α (pink), β (violet), γ (magenta), δ (purple) and DPE phase α (green) (*cf*. Fig. 5).

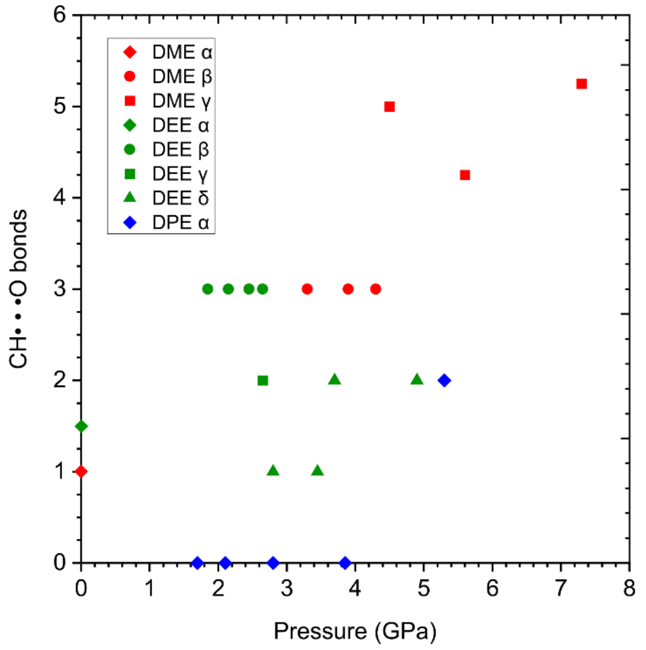

Figure S24. The number of H-donors accepting the O-atoms within the hydrogen bonds CH···O (per one molecule in the unit cell) in different phases of DME, DEE and DPE.

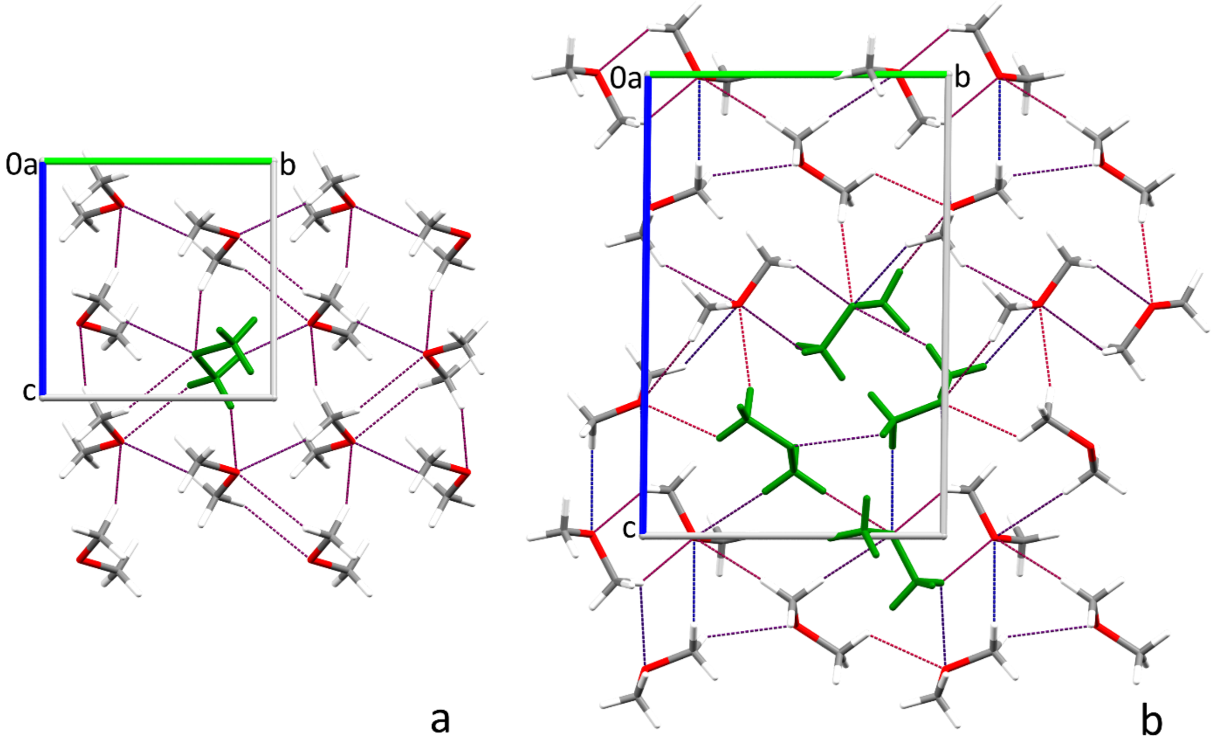

Figure S25. CH···O hydrogen bonding patterns in the crystal structures of DME polymorphs (a) β and (b) γ. The symmetry independent molecules are indicated in green, whereas the CH···O contacts are marked by dotted lines.

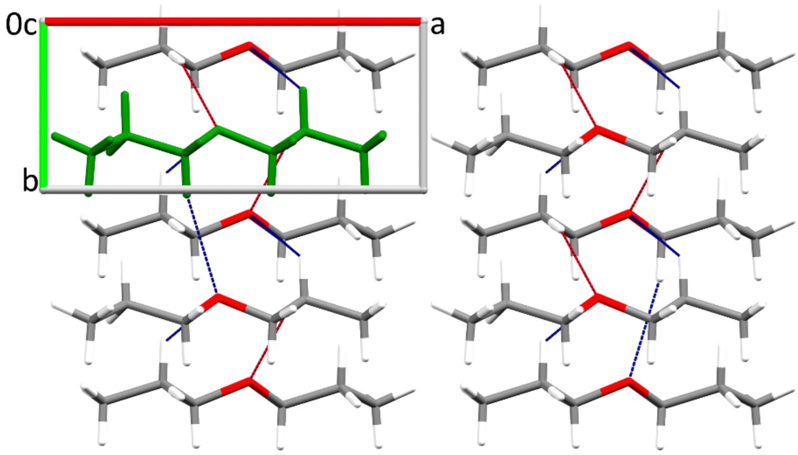

Figure S26. CH···O hydrogen bonding patterns in the high-pressure crystal structure of DPE. The symmetry independent molecule is indicated in green, whereas the CH···O contacts are marked by dotted lines.

Table S1. Crystal data and structure refinement details of DME phase β at 3.30, 3.90, 4.30 GPa and phase γ at 4.50, 5.60, 7.30 GPa (all at 295 K).

	C_2H_6O phase β	C_2H_6O phase β	C_2H_6O phase β	C_2H_6O phase γ	C_2H_6O phase γ	C_2H_6O phase γ
Pressure (GPa)	3.30(2)	3.90(2)	4.30(2)	4.50(2)	5.60(2)	7.30(2)
Temperature (K)	295(2)	295(2)	295(2)	295(2)	295(2)	295(2)
Formula weight	46.07	46.07	46.07	46.07	46.07	46.07
Crystal colour	colourless	colourless	colourless	colourless	colourless	colourless
Crystal size (mm)	0.30x0.29x0.25	0.33x0.33x0.24	0.38x0.38x0.23	0.34x0.32x0.22	0.37x0.35x0.21	0.40x0.38x0.20
Crystal system	monoclinic	monoclinic	monoclinic	triclinic	triclinic	triclinic
Space group	$P2_1/c$	$P2_1/c$	$P2_1/c$	$P\bar{1}$	$P\bar{1}$	$P\bar{1}$
Unit cell dimensions						
a (Å)	5.5541(4)	5.5277(4)	5.5073(4)	4.3394(12)	4.2888(17)	4.250(4)
b (Å)	6.6179(11)	6.527(10)	6.493(5)	8.414(2)	8.3045(18)	8.198(3)
c (Å)	6.964(3)	6.8941(5)	6.8431(8)	12.821(6)	12.7912(13)	12.6105(19)
α (°)	90	90	90	90.55(4)	90.249(12)	90.641(19)
β (°)	103.835(19)	103.869(6)	103.848(8)	93.89(6)	93.92(2)	93.97(3)
γ (°)	90	90	90	90.83(2)	90.14(3)	90.04(5)
Volume ($Å^3$)	248.56(12)	241.5(4)	237.58(19)	467.0(3)	454.5(2)	438.3(4)
Z, Z′	4, 1	4, 1	4, 1	8, 4	8, 4	8, 4
V/Z	62.14(3)	60.375(1)	59.395(5)	58.375(4)	56.8125(3)	54.7875(5)
D_x ($g·cm^{-3}$)	1.231	1.267	1.288	1.311	1.346	1.396
Wavelength MoKα, λ (Å)	0.71073	0.71073	0.71073	0.71073	0.71073	0.71073
Absorption coefficient (mm^{-1})	0.095	0.098	0.100	0.101	0.104	0.108
F(000) (e)	104	104	104	208	208	208
2Θ max (°)	51.82	51.45	52.24	52.31	52.51	52.24
Min./Max. indices *h*, *k*, *l*	-6/6, -7/7, -4/4	-6/6, -1/1, -8/8	-6/6, -3/3, -7/7	-5/5, -10/10, -5/6	-2/2, -10/9, -15/15	-2/2, -9/9, -15/15
Reflections collected/unique	850/147	634/107	721/129	2659/556	2631/559	2349/523
R_{int} (all data)	0.0646	0.0308	0.0295	0.0338	0.0306	0.0418
Observed reflections ($F^2 > 2\sigma(F^2)$)	132	99	118	481	486	389
Data/restraints/parameters	147/0/31	107/0/15	129/0/31	556/0/117	559/0/58	523/0/118
Goodness of fit on F^2	1.204	1.192	1.181	1.131	1.135	1.055
Final R_1 indices ($F^2 > 2\sigma(F^2)$)	0.0708	0.0690	0.0525	0.0554	0.1173	0.0793
R_1/wR_2 indices (all data)	0.0776/0.1927	0.0728/0.1724	0.0566/0.1399	0.0628/0.1553	0.1296/0.2604	0.1062/0.1868
$\Delta\sigma_{max}$, $\Delta\sigma_{min}$ ($eÅ^{-3}$)	0.19/-0.17	0.17/-0.12	0.14/-0.11	0.15/-0.14	0.23/-0.19	0.15/-0.14
Weighting scheme: x; y[a]	0.1102;0.24	0.1005/0.17	0.1045/0.04	0.0885/0.32	0.0699/2.71	0.0557/1.68

[a] $w=1/(\sigma^2(Fo^2)+x^2P^2+yP)$, where $P=(\mathrm{Max}(Fo^2,0)+2Fc^2)/3$

Table S2. Crystal data and structure refinement details of DEE phase β at 1.85, 2.15, 2.45, 2.65 GPa and phase γ at 2.65 GPa (all at 295 K).

	$C_4H_{10}O$ phase β	$C_4H_{10}O$ phase β	$C_4H_{10}O$ phase β	$C_4H_{10}O$ phase β	$C_4H_{10}O$ phase γ
Pressure (GPa)	1.85(2)	2.15(2)	2.45(2)	2.65(2)	2.65(2)
Temperature (K)	295(2)	295(2)	295(2)	295(2)	295(2)
Formula weight	74.12	74.12	74.12	74.12	74.12
Crystal colour	colourless	colourless	colourless	colourless	colourless
Crystal size (mm)	0.37x0.36x0.27	0.38x0.34x0.26	0.39x0.36x0.25	0.40x0.37x0.24	0.40x0.36x0.24
Crystal system	monoclinic	monoclinic	monoclinic	monoclinic	monoclinic
Space group	$P2_1/c$	$P2_1/c$	$P2_1/c$	$P2_1/c$	$I2/a$
Unit cell dimensions					
a (Å)	6.8268(3)	6.7948(3)	6.7759(3)	6.7612(3)	7.7073(12)
b (Å)	8.1428(17)	8.0952(12)	8.0552(14)	8.0271(15)	4.0885(4)
c (Å)	7.7731(3)	7.7259(2)	7.6873(2)	7.6687(3)	13.233(2)
β (°)	93.443(4)	93.621(3)	93.713(3)	93.784(4)	93.793(16)
Volume (Å^3)	431.32(9)	424.12(7)	418.70(8)	415.29(8)	416.07(10)
Z, *Z'*	4, 1	4, 1	4, 1	4, 1	4, 0.5
V/Z	107.83(2)	106.03(2)	104.675(2)	103.8225(2)	104.0175(3)
D_x (g·cm^{-3})	1.141	1.161	1.176	1.185	1.183
Wavelength MoKα, λ (Å)	0.71073	0.71073	0.71073	0.71073	0.71073
Absorption coefficient (mm^{-1})	0.079	0.080	0.081	0.082	0.081
F(000) €	168	168	168	168	168
2Θ max (°)	52.79	53.27	52.89	52.79	52.71
Min./Max. indices *h*, *k*, *l*	-8/8, -5/5, -9/9	-8/8, -5/5, -9/9	-8/8, -5/5, -9/9	-8/7, -5/5, -9/9	-7/8, -4/4, -14/14
Reflections collected/unique	2198/386	2208/386	2154/377	2120/375	1160/223
R_{int} (all data)	0.0239	0.0250	0.0245	0.0216	0.0131
Observed reflections ($F^2 > 2\sigma(F^2)$)	352	353	346	342	202
Data/restraints/parameters	386/0/48	386/0/48	377/0/48	375/0/48	223/0/25
Goodness of fit on F^2	1.121	1.168	1.138	1.088	1.130
Final R_1 indices ($F^2 > 2\sigma(F^2)$)	0.0353	0.0327	0.0390	0.0388	0.0371
R_1/wR_2 indices (all data)	0.0403/0.0988	0.0367/0.0928	0.0421/0.1110	0.0423/0.1110	0.0417/0.1085
$\Delta\sigma_{max}$, $\Delta\sigma_{min}$ (eÅ^{-3})	0.10/-0.11	0.09/-0.09	0.12/-0.12	0.14/-0.15	0.09/-0.11
Weighting scheme: x; y[a]	0.0644;0.04	0.0569/0.03	0.0773/0.03	0.0819/0.03	0.0545/0.17

[a] $w=1/(\sigma^2(Fo^2)+x^2P^2+yP)$, where $P=(\text{Max}(Fo^2,0)+2Fc^2)/3$

Table S3. Crystal data and structure refinement details of DEE phase δ at 2.80, 3.45, 3.70, 4.90 GPa (all at 295 K).

	$C_4H_{10}O$ phase δ	$C_4H_{10}O$ phase δ	$C_4H_{10}O$ phase δ	$C_4H_{10}O$ phase δ
Pressure (GPa)	2.80(2)	3.45(2)	3.70(2)	4.90(2)
Temperature (K)	295(2)	295(2)	295(2)	295(2)
Formula weight	74.12	74.12	74.12	74.12
Crystal colour	colourless	colourless	colourless	colourless
Crystal size (mm)	0.36x0.36x0.23	0.37x0.37x0.22	0.38x0.38x0.21	0.40x0.40x0.20
Crystal system	triclinic	triclinic	triclinic	triclinic
Space group	$P\bar{1}$	$P\bar{1}$	$P\bar{1}$	$P\bar{1}$
Unit cell dimensions				
a (Å)	5.1196(4)	5.0809(5)	5.072(3)	5.0182(19)
b (Å)	5.6659(10)	5.6329(10)	5.629(2)	5.575(3)
c (Å)	7.2999(4)	7.2451(10)	7.215(5)	7.136(3)
α (°)	97.275(8)	97.382(13)	97.43(5)	97.36(4)
β (°)	102.728(6)	102.749(10)	102.91(6)	102.81(3)
γ (°)	96.747(10)	97.002(11)	97.15(5)	97.72(4)
Volume (Å^3)	202.56(4)	198.13(5)	196.6(2)	190.31(14)
Z, *Z'*	2, 1	2, 1	2, 1	2, 1
V/Z	101.28(2)	99.065(3)	98.3(1)	95.155(7)
D_x (g·cm^{-3})	1.215	1.242	1.252	1.293
Wavelength MoKα, λ (Å)	0.71073	0.71073	0.71073	0.71073
Absorption coefficient (mm^{-1})	0.084	0.086	0.086	0.089
F(000) (e)	84	84	84	84
2Θ max (°)	52.25	52.25	55.46	55.72
Min./Max. indices *h*, *k*, *l*	-6/6, -4/4, -8/8	-6/6, -5/4, -8/8	-5/5, -7/7, -7/7	-5/5, -6/6, -8/8
Reflections collected/unique	1217/244	1179/243	1025/199	944/195
R_{int} (all data)	0.0138	0.0183	0.0383	0.0950
Observed reflections ($F^2 > 2\sigma(F^2)$)	225	218	177	153
Data/restraints/parameters	244/0/48	243/0/23	199/0/23	195/0/23
Goodness of fit on F^2	1.179	1.788	1.166	1.086
Final R_1 indices ($F^2 > 2\sigma(F^2)$)	0.0388	0.1011	0.0489	0.0610
R_1/wR_2 indices (all data)	0.0419/0.1000	0.1071/0.3608	0.0553/0.1407	0.0739/0.1816
$\Delta\sigma_{max}$, $\Delta\sigma_{min}$ (eÅ^{-3})	0.09/-0.08	0.30/-0.29	0.09/-0.10	0.12/-0.12
Weighting scheme: x; y^a	0.0403/0.09	0.2000/0	0.0907/0.03	0.1287/0.01

$^a w=1/(\sigma^2(Fo^2)+x^2P^2+yP)$, where $P=(\mathrm{Max}(Fo^2,0)+2Fc^2)/3$

Table S4. Crystal data and structure refinement details of DPE phase α at 1.70, 2.10, 2.80, 3.85, 5.30 GPa (all at 295 K).

	**$C_6H_{14}O$ phase α**	**$C_6H_{14}O$ phase α**	**$C_6H_{14}O$ phase α**	**$C_6H_{14}O$ phase α**	**$C_6H_{14}O$ phase α**
Pressure (GPa)	1.70(2)	2.10(2)	2.80(2)	3.85(2)	5.30(2)
Temperature (K)	295(2)	295(2)	295(2)	295(2)	295(2)
Formula weight	102.18	102.18	102.18	102.18	102.18
Crystal colour	colourless	colourless	colourless	colourless	colourless
Crystal size (mm)	0.39x0.37x0.25	0.39x0.38x0.24	0.46x0.43x0.23	0.38x0.36x0.22	0.44x0.40x0.21
Crystal system	monoclinic	monoclinic	monoclinic	monoclinic	monoclinic
Space group	$P2_1/c$	$P2_1/c$	$P2_1/c$	$P2_1/c$	$P2_1/c$
Unit cell dimensions					
a (Å)	9.416(4)	9.368(8)	9.293(7)	9.198(9)	9.099(12)
b (Å)	4.1817(3)	4.1229(4)	4.0686(2)	4.0064(3)	3.9465(3)
c (Å)	15.579(7)	15.434(4)	15.1855(10)	14.9459(15)	14.649(8)
β (°)	101.23(5)	100.84(6)	100.35(3)	99.54(4)	98.32(10)
Volume (Å^3)	601.7(4)	585.5(6)	564.8(4)	543.1(5)	520.5(7)
Z, *Z′*	4, 1	4, 1	4, 1	4, 1	4, 1
V/*Z*	150.425(1)	146.375(1)	141.2(1)	135.775(1)	130.125(2)
D_x (g·cm^{-3})	1.128	1.159	1.202	1.249	1.304
Wavelength MoKα, λ (Å)	0.71073	0.71073	0.71073	0.71073	0.71073
Absorption coefficient (mm^{-1})	0.073	0.075	0.078	0.081	0.085
F(000) (e)	232	232	232	232	232
2Θ max (°)	52.86	53.06	53.10	52.91	52.55
Min./Max. indices *h*, *k*, *l*	-8/8, -5/5, -14/14	-6/6, -5/5, -16/16	-4/4, -5/5, -19/19	-4/4, -5/5, -18/18	-7/7, -4/4, -16/16
Reflections collected/unique	3001/386	2850/383	2881/356	2785/347	2594/310
R_{int} (all data)	0.0254	0.0297	0.0270	0.0297	0.0350
Observed reflections ($F^2 > 2\sigma(F^2)$)	286	287	282	281	232
Data/restraints/parameters	386/0/66	383/0/66	356/0/66	347/0/66	310/6/66
Goodness of fit on F^2	1.077	1.053	1.082	1.111	1.060
Final R_1 indices ($F^2 > 2\sigma(F^2)$)	0.0455	0.0524	0.0456	0.0308	0.0469
R_1/wR_2 indices (all data)	0.0661/0.1513	0.0723/0.1416	0.0591/0.1447	0.0424/0.0900	0.0666/0.1229
$\Delta\sigma_{max}$, $\Delta\sigma_{min}$ (eÅ^{-3})	0.10/-0.09	0.10/-0.10	0.09/-0.12	0.05/-0.06	0.10/-0.08
Weighting scheme: x; y[a]	0.1115/0.01	0.0676/0.31	0.0950/0.11	0.0571/0.02	0.0445/0.34

[a] $w=1/(\sigma^2(Fo^2)+x^2P^2+yP)$, where $P=(\mathrm{Max}(Fo^2,0)+2Fc^2)/3$
